# Risk factors for diabetes mellitus after acute pancreatitis: a systematic review and meta-analysis

**DOI:** 10.3389/fmed.2023.1257222

**Published:** 2024-01-09

**Authors:** Olga Julia Zahariev, Stefania Bunduc, Adrienn Kovács, Dóra Demeter, Luca Havelda, Bettina Csilla Budai, Dániel Sándor Veres, Nóra Hosszúfalusi, Bálint Mihály Erőss, Brigitta Teutsch, Márk Félix Juhász, Péter Hegyi

**Affiliations:** ^1^Centre for Translational Medicine, Semmelweis University, Budapest, Hungary; ^2^Institute of Pancreatic Diseases, Semmelweis University, Budapest, Hungary; ^3^Institute for Translational Medicine, Medical School, University of Pécs, Pécs, Hungary; ^4^Carol Davila University of Medicine and Pharmacy, Bucharest, Romania; ^5^Department of Internal Medicine and Hematology, Semmelweis University, Budapest, Hungary; ^6^Dietetic Services, Central Hospital of Northern Pest - Military Hospital, Budapest, Hungary; ^7^Department of Biophysics and Radiation Biology, Semmelweis University, Budapest, Hungary; ^8^Heim Pál National Pediatric Institute, Budapest, Hungary; ^9^Translational Pancreatology Research Group, Interdisciplinary Center of Excellence for Research Development and Innovation University of Szeged, Szeged, Hungary

**Keywords:** diabetes mellitus, prediabetes, acute pancreatitis (AP), pancreatitis—complications, gastrointestinal disorders, risk factor (RF)

## Abstract

**Introduction:**

Within 5 years of having acute pancreatitis (AP), approximately 20% of patients develop diabetes mellitus (DM), which later increases to approximately 40%. Some studies suggest that the prevalence of prediabetes (PD) and/or DM can grow as high as 59% over time. However, information on risk factors is limited. We aimed to identify risk factors for developing PD or DM following AP.

**Methods:**

We systematically searched three databases up to 4 September 2023 extracting direct, within-study comparisons of risk factors on the rate of new-onset PD and DM in AP patients. When PD and DM event rates could not be separated, we reported results for this composite outcome as PD/DM. Meta-analysis was performed using the random-effects model to calculate pooled odds ratios (OR) with 95% confidence intervals (CI).

**Results:**

Of the 61 studies identified, 50 were included in the meta-analysis, covering 76,797 participants. The studies reported on 79 risk factors, and meta-analysis was feasible for 34 risk factor and outcome pairs. The odds of developing PD/DM was significantly higher after severe and moderately severe AP (OR: 4.32; CI: 1.76–10.60) than mild AP. Hypertriglyceridemic AP etiology (OR: 3.27; CI: 0.17–63.91) and pancreatic necrosis (OR: 5.53; CI: 1.59–19.21) were associated with a higher risk of developing PD/DM. Alcoholic AP etiology (OR: 1.82; CI: 1.09–3.04), organ failure (OR: 3.19; CI: 0.55–18.64), recurrent AP (OR: 1.89; CI: 0.95–3.77), obesity (OR: 1.85; CI: 1.43–2.38), chronic kidney disease (OR: 2.10; CI: 1.85–2.38), liver cirrhosis (OR: 2.48; CI: 0.18–34.25), and dyslipidemia (OR: 1.82; CI: 0.68–4.84) were associated with a higher risk of developing DM.

**Discussion:**

Severe and moderately severe AP, alcoholic and hypertriglyceridemic etiologies, pancreatic necrosis, organ failure, recurrent acute pancreatitis and comorbidities of obesity, chronic kidney disease liver disease, and dyslipidemia are associated with a higher risk of developing PD or DM.

**Systematic review registration:**

https://www.crd.york.ac.uk/prospero/, identifier CRD42021281983.

## 1 Introduction

Acute pancreatitis (AP) is characterized by premature activation of pancreatic enzymes leading to autodigestion and inflammation of the pancreatic tissue. Potential short-term complications include acute pancreatic fluid collection, pancreatic necrosis, and organ failure (1). Patients with preexisting diabetes mellitus (DM) have an increased risk of developing complications during an AP episode (2). Additionally, elevated glucose levels during hospitalization are associated with more severe AP episodes and increased mortality rates (3). Moreover, it is gaining recognition that DM might also develop after AP as a potential long-term complication (4, 5).

A large population-based study of 14,830 people found that compared to the general population the risk of DM is 2-fold having had a single episode of mild AP (6). Multiple meta-analyses found that within 5 years of an AP episode 18–20% of the patients develop DM, which later increases to approximately 37–40% (7, 8). New-onset prediabetes (PD) is also frequent. Das et al. found the combined incidence of PD and DM to be 35% in the first year following the first AP episode, increasing to 59% after 5 years (7). Not only is the risk of these conditions substantially increased in the context of AP, but their therapy is also challenging. Post-AP DM is recognized as a distinct subtype of DM (9) with more frequent hypoglycemic events (10, 11) and simultaneously greater insulin needs (5, 12, 13) than type 2 DM.

Studies focusing on acute pancreatitis patients with extended follow-up periods are limited (14) and investigations into the implications of developing post-AP DM are even more scarce. Compared to type 2 DM, post-AP DM carries a higher risk of cardiovascular and cerebrovascular disease based on cohort studies exceeding 150,000 patients (5, 11). A population-based matched cohort study of 10,549 individuals in New Zealand reported higher cancer-related deaths (not including pancreatic cancer) and increased mortality from gastrointestinal and infectious diseases in patients with post-AP DM compared to type 2 DM (15). Patients with post-AP DM also have an increased risk of all-cause mortality compared to patients with type 2 DM (5, 10, 11).

Therefore, it is essential to understand the risk factors of developing PD and DM after AP, to facilitate prompt diagnosis and treatment. Two previous meta-analyses provided data on possible risk increasing features, but with conflicting results (7, 8). One possible reason is that instead of pooling direct within-study comparisons these studies used analytical methods conferring a significantly higher risk of bias and less accurate estimations, i.e., meta-regression of PD and DM based on the proportion of a proposed risk factor, indirect comparison of PD and DM prevalence in individuals with different proposed risk factors. The number of analyzed variables was also very limited (to severity, alcoholic and biliary etiology, necrosis, age, sex, follow-up length, and publication year).

We aimed to conduct a comprehensive systematic review and meta-analysis of all available risk factors for PD and DM development after AP, including only studies where prognostic factors are directly compared, allowing for more reliable conclusions.

## 2 Methods

### 2.1 Protocol and reporting

Our review followed the Cochrane Handbook for Systematic Reviews (16) and adhered to the Preferred Reporting Items for Systematic Reviews and Meta-Analyses (PRISMA) 2020 guideline (Supplementary Table S1) (17). The study protocol was registered with the International Prospective Register of Systematic Reviews (PROSPERO, CRD42021281983).

### 2.2 Eligibility criteria

Our study aimed to investigate risk factors for developing PD and DM following AP, via analyzing all factors assessed during the hospitalization with AP, that were compared between new-onset PD or DM and normal glucose regulation groups. To establish the eligibility criteria, we used the PECOTS framework.

Population (P): adult AP patients without confirmed DM at discharge. Exposure and comparator (E): any factor assessed at the time of hospitalization with AP and (C) its control group, such as severe vs. non-severe AP, necrosis vs. absence of necrosis, smoking vs. not smoking, male vs. female. Classification of AP severity has changed over the years. Our study group’s data in two ways firstly comparing severe AP (SAP) vs. moderately severe and mild AP as one group and alternatively comparing SAP and moderately severe AP as one group vs. mild AP. Some studies applied classification criteria with only two categories: severe and non-severe AP. These studies defined SAP based on 1992 Atlanta criteria (18–21), Scoring ≥8 on APACHE II (22), ≥3 Ranson score (23), and ≥ 2 Japanese severity score (24). We analyzed the findings of these studies using the categories of SAP vs. moderate and mild AP as one group.

Outcome (O): Number of AP patients who developed, after hospital discharge: DM or PD (impaired fasting glucose, impaired glucose tolerance, and HbA1c ≥5.7 and < 6.5%) as reported by the study authors. Multiple studies provided the number of patients who developed PD or DM combined; we included this composite outcome in our analyses as PD/DM. In case of studies providing incomplete or no definition for glycemic outcomes or not stating explicitly that preexisting DM was excluded from the cohort, this uncertainty was taken into account during the risk of bias assessment.

Timing (T): Initially, we planned to include studies assessing the outcome at least 3 months after hospital discharge. However, we decided to deviate and include all studies that reported on the relevant outcomes after hospital discharge because of the limited and heterogeneous data on follow-up and diagnosis time intervals.

Study design (S): The analysis included interventional and observational studies that met the criteria of our review’s PECO framework. Case reports, case series, and studies with less than 10 participants per outcome group or less than 10 participants in the exposed or comparator group were excluded. Conference abstracts were retained.

### 2.3 Search strategy and selection process

The systematic search was carried out in three databases: MEDLINE (via PubMed), Embase, and Cochrane Central Register of Controlled Trials (CENTRAL) from database inception to September 04, 2023 without any filters or restrictions. The main concepts in the search strategy were prediabetes, diabetes, acute, and pancreatitis. See Supplementary Table S2 for the detailed search key and selection process.

### 2.4 Data collection process and data items

Data collection process is detailed in Supplementary Table S2. Data on the following variables were collected when available: country, year of publication, study period, follow-up time, name and the number of centers, study design, sample size, age, sex and weight of participants, inclusion and exclusion criteria of participants, classification of AP severity, outcome domains reported and their assessment method, and risk factors during the initial AP episode and their definitions. For a complete list of the risk factors investigated in relation to new-onset PD, DM, or PD/DM by the included studies, see Supplementary Table S3.

### 2.5 Data synthesis

We calculated odds ratios (ORs) with 95% confidence intervals (CI). Refer to Supplementary Table S4 for detailed description.

### 2.6 Risk of bias

Two independent reviewers (OZ and AK) assessed each study for risk of bias using the Quality In Prognosis Studies (QUIPS) tool (25). Disagreements were resolved by discussion until reaching a consensus. Risk of bias analyses were conducted for each outcome and prognostic factor separately. To simplify and ease the interpretation of these results, three summary Risk of bias assessments were created for the three main outcomes (DM, PD, and PD/DM), taking into account the worst possible scenario for each study and each domain.

### 2.7 Publication bias

To assess the possibility of publication bias (small study effect), we created and visually assessed funnel plots for every analysis where at least six studies were included. Harbord modified Egger’s test was performed in the case of 10 or more included studies (26), with a *p* < 0.1 indicating statistical significance for funnel plot asymmetry.

## 3 Results

### 3.1 Study selection

**Figure 1:**
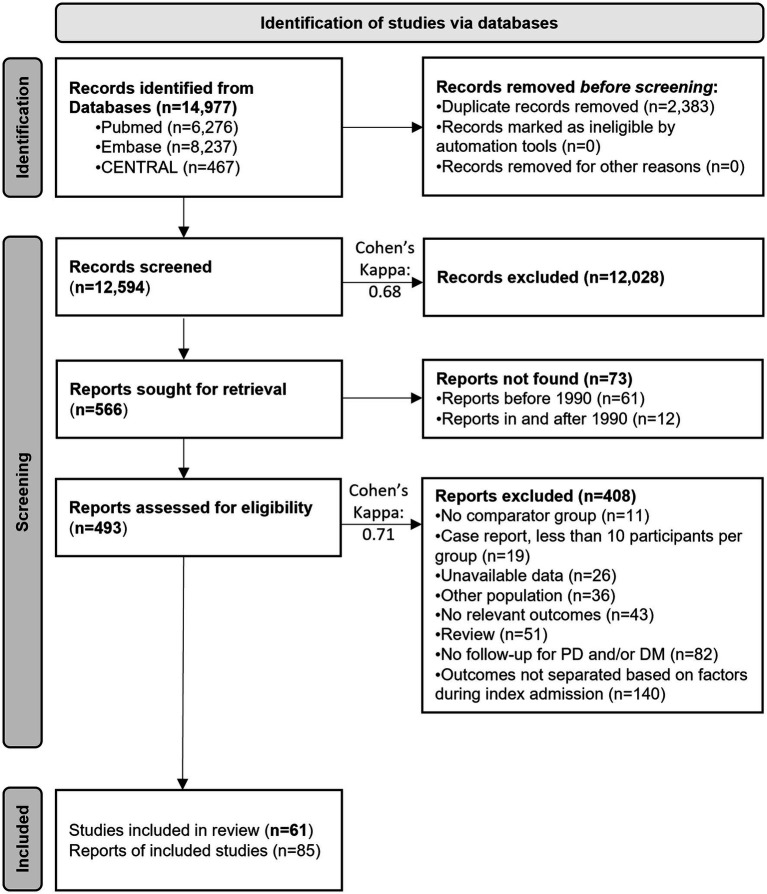
PRISMA flow diagram of screened and included studies. PD, Prediabetes; DM, Diabetes mellitus.

The systematic search yielded 14,977 results (Figure 1). Overall, 61 studies with 85 reporting articles were eligible for inclusion. The meta-analysis encompassed 50 studies and 76,797 patients.

### 3.2 Characteristics of included studies

Key study characteristics are summarized in Table 1. Approximately 68% of the studies were based on the general AP population, four included only SAP patients, six focused on necrotizing AP patients and in 10 studies participants were superselected for other criteria. The outcome was reported as PD, DM, and PD/DM in 6, 43, and 22 studies, respectively. A total of 79 prognostic factors were reported on by at least one study and the unique combinations of prognostic factors and outcomes numbered 137 different comparisons. Meta-analysis was possible in the case of 34 risk factor and outcome pairs.

**Table 1:** Basic characteristics of the included studies.

Study identifier	Country	Study design	Population	Total No. of participants (male %)	Age ^*^ (year)	Outcome type			Outcome assessment method	Mean time to follow-up (months)^*^
						PD	DM	PD/DM		
Akbar et al. (48)^†^	India	Prospective cohort	AP	86 (77%)	36^§^ ±12^¶^	23.3%	10.5%	33.7%	FPG, OGTT, HbA1c	12^§§^
Akbar et al. (32)	India	Prospective cohort	AP	86 (77%)	33^§^ (26–44.2)^||^	23.3%	10.5%	33.7%	FPG, OGTT, HbA1c	12^§§^
Andersson et al. (18)	Sweden	Prospective cohort	AP	40 (40%)	61^§^ (48-68)^||^	33.3%	23.1%	56.4%	FPG, OGTT	42 (36–53)
Angelini et al. (53)	Italy	Prospective cohort	ANP	27 (89%)	NA	44.4%	14.8%	59.3%	OGTT	12–36^||^
Bharmal et al. (71)^‡^	New Zealand	Cross-sectional	AP	79 (62%)	50 (41–63)	34.2%	NA	NA	FPG, HbA1c	26 (6–47)
Bharmal et al. (50)	New Zealand	Prospective cohort	AP	120 (58%)	G1: 48 ± 16 ; G2: 54 ± 16 ; G3: 53 ± 20	NA	6.6%	NA	HbA1c	24^§§^
Bharmal et al. (72)^‡^	New Zealand	Prospective cohort	AP	68 (47%)	G1: 60 ± 20 ; G2: 55 ± 18 ; G3: 48 ± 15	20.5%	NA	NA	FPG, HbA1c	24 §§
Bojková et al. (55)	Czech Republic	Retrospective cohort	AP progressing to CP in 1–2 years	56 (52%)	52**	NA	21.4%	NA	NA	12–24^||^
Boreham and Ammori (61)	United Kingdom	Prospective cohort	AP	23 (57%)	55 (21–77)	NA	17.4%	NA	FPG	3^§§^
Burge and Gabaldon-Bates (69)	New Mexico	Retrospective cohort	AP	887 (56%)	NA	NA	11.0%	NA	Diagnostic codes	NA
Buscher et al. (57)	Netherlands	Prospective case–control	ANP	20 (75%)	52^**^ ±3^††^	30.0%	25.0%	55.0%	OGTT	63^**^ (8–136)^||^
Castoldi et al. (19)	Italy	Cross-sectional	AP	631 (50%)	61 ± 19	NA	3.5%	NA	Questionnaire	52 ± 8
Chandrasekaran et al. (73)^‡^	India	Prospective cohort	SAP	35 (83%)	37§ ±10^¶^	NA	48.6%	NA	OGTT	26 ± 18
Cho et al. (42)	New Zealand	Retrospective cohort	AP with gout	9,471 (48%)	56 ± 19	NA	5.9%	NA	Diagnostic codes Medication prescription	46 ± 34
Cho et al. (64)	New Zealand	Retrospective cohort	MAP, MSAP	10,870 (49%)	56 ± 19	NA	6.5%	NA	Diagnostic codes, medication prescription	G1: 107 ± 0.4 ; G2: 95 ± 0.6
Chowdhury et al. (38)^†^	USA	Prospective cohort	AP	723 (50.2%)	43 ± 14	NA	4.6%	NA	HbA1c	9–63^||^
Doepel et al. (56)	Finland	Prospective cohort	SAP	37 (68%)	49^**^ (26-90)^||^	10.8%	54.1%	64.9%	FPG, OGTT, and HbA1c	74^**^ (12–168)^||^
Ermolov et al. (74)^‡^	Russia	Prospective cohort	ANP	210 (69%)	55 ± 13	NA	29.5%	NA	FPG	102 ± 36
Firkins et al. (43)	United States	Retrospective case–control	AP	42,818 (47%)	53** ±0.2^‡‡^	NA	5.9%	NA	Diagnostic code	12^§§^
Frey et al. (54)	United States	Retrospective cohort	AP	306 (69%)	NA	NA	24.8%	NA	Medication prescription	NA
Garip et al. (22)	Turkey	Prospective cohort	AP	109 (53%)	57 ± 16	NA	NA	34.4%	OGTT	32^**^ (6-48)^||^
Gold-Smith et al. (39)	New Zealand	Cross-sectional	AP non-iatrogenic	93 (61%)	53 (42–65)	NA	12.9%	NA	FPG, HbA1c	22 (7–46)
Guo et al. (70)	China	Retrospective cohort	AP	492 (64%)	G1: 44 (35–54) ; G2: 52 (39–63)	NA	NA	31.0%	FPG, OGTT, HbA1c, random blood glucose	3–60^||^
Halonen et al. (52)	Finland	Prospective cohort	SAP	145 (83%)	44^**^ (20-78)^||^	NA	41.4%	NA	Medical records and questionnaire	66 ± 32
Hietanen et al. (63)	Finland	Prospective cohort	AP	62 (84%)	G1: 49§ (21-73)^||^ ; G2: 55§ (27-80) ^||^	NA	8.1%	NA	NA	31^§^ (17-53)^||^
Ho et al. (20)	Taiwan	Retrospective cohort	AP	12,284 (71%)	NA	NA	5.0%	NA	Diagnostic codes	12–120^||^
Hochman et al. (60)	Canada	Prospective cohort	SAP	25 (64%)	59^**^ (37-86)^||^	NA	32.0%	NA	Questionnaire	24–36^||^
Huang et al. (75)^‡^	China	Prospective cohort	ANP	50 (52%)	G1: 53 ± 16 ; G2: 51 ± 15	NA	Not stated	NA	FPG, random blood glucose	3–69^||^
Koziel et al. (44)	Poland	Prospective cohort	MAP, SAP	150 (63%)	G1: 52 ± 17 ; G2: 57 ± 16	NA	13.5%	NA	HbA1c	G1: 14 ± 4 ; G2: 15 ± 4
Li et al. (47)	New Zealand	Cross-sectional	AP non-iatrogenic	72 (67%)	G1: 60 (47–67) ; G2: 51 (43–59)	NA	NA	50.0%	FPG, HbA1c	27^**^ ±2^‡‡^
Lv et al. (37)	China	Retrospective cohort	AP	1,804 (63%)	48 (36–62)	NA	6.1%	NA	Questionnaire	37 (21–54)
Ma et al. (45)	China	Cross-sectional	AP non-iatrogenic	616 (63%)	47 (37–63)	NA	20.0%	NA	OGTT, HbA1c	3^§§^
Malecka-Panas et al. (67)	Poland	Prospective cohort	Alcoholic AP with pseudocyst	50 (68%)	46 ± 14	NA	NA	26.0%	OGTT	46 ± 20
Malecka-Panas et al. (23)	Poland	Prospective cohort	AP BMI ≤25 kg/m^2^	82 (67%)	47 ± 8	4.9%	15.9%	20.3%	OGTT	56 ± 43
Man et al. (41)	Romania	Prospective cohort	AP	308 (54%)	G1: 60 § (18-90)^||^ G2: 45.5 § (40-65)^||^	NA	2.5%	NA	FPG, OGTT	12^§§^
Miko et al. (28)^†^	Hungary	Prospective cohort	AP	178 (NA)	NA	34.3%	15.7%	50.0%	OGTT	12^§§^
Nikkola et al. (29)	Finland	Prospective cohort	Alcoholic AP	77 (90%)	48^§^ (25-71)^||^	19.1%	19.1%	38.2%	FPG, OGTT, HbA1c	126^§^ (37–155)^||^
Nikolic et al. (76)^‡^	Sweden, Italy	Retrospective cohort	AP	35 (48.6%)	41 § (26-NA)^||^	NA	8.6%	NA	Diagnostic codes, medical records	54^§^
Norbitt et al. (65)	New Zealand	Cross-sectional	AP	69 (59.4%)	NA	NA	NA	53.6%	FPG, HbA1c	60^§§^
Norbitt et al. (77)^‡^	New Zealand	Cross-sectional	AP	69 (59.4%)	NA	NA	NA	53.6%	FPG, HbA1c	NA
Patra and Das (40)	India	Retrospective cohort	AP	100 (64%)	42^**^ (14-88)^||^	NA	17.0%	NA	FPG, OGTT	60^§§^
Pendharkar et al. (66)	New Zealand	Cross-sectional	AP non-iatrogenic	83 (60%)	G1: 47 ± 15 ; G2: 57 ± 13	NA	NA	36.1%	FPG, HbA1c	G1: 33 ± 30 ; G2: 23 ± 19
Pendharkar et al. (33)	New Zealand	Cross-sectional	AP non-iatrogenic	83 (60%)	NA	NA	NA	36.1%	FPG, HbA1c	30^**^
Robertson et al. (36)	UK	Prospective cohort	AP	337 (60%)	G1: 57 (17–90) G2: 58.5 (21–84)	NA	11.2%	NA	Insulin prescription	22 § (11-33)^||^
Symersky et al. (21)	Netherlands	Prospective cohort	biliary and iatrogenic AP	34 (47%)	53^**^ ±3^‡‡^	NA	35.3%	NA	OGTT	55^**^ (12-90)^||^
Takeyama (24)	Japan	Retrospective cohort	MSAP, SAP	714 (NA)	NA	NA	13.0%	NA	FPG	≥ 156
Thiruvengadam et al. (78)^‡^	USA	Retrospective cohort	AP	118,479 (NA)	NA	NA	10.6%	NA	Diagnostic codes, medication prescription	42^§^
Trgo et al. (34)	Croatia	Prospective cohort	MAP, MSAP	33 (100%)	NA	NA	NA	42.4%	OGTT	1^§§^
Trikudanathan et al. (79)^†‡^	USA	Prospective cohort	ANP	390 (66%)	51 (36–64)	NA	25.8%	NA	NA	13 (3–35)
Tu et al. (30)	China	Prospective cohort	AP	113 (66%)	47** ±1^‡‡^	29.2%	30.1%	59.3%	OGTT, HbA1c	43 ± 4
Tu et al. (46)	China	Prospective cohort	AP	256 (66%)	44** ±1^‡‡^	NA	NA	60.2%	FPG, random blood glucose, OGTT	43 ± 4
Tu et al. (4)	China	Cross-sectional	AP	88 (NA)	NA	NA	25.0%	NA	FPG, OGTT, HbA1c	6–90^||^
Uomo et al. (68)	Italy	Prospective cohort	ANP	40 (43%)	48 ± 18	NA	15.8%	NA	FPG, OGTT	180 ± 13
Vujasinovic et al. (35)	Slovenia	Prospective cohort	AP developing PEI	21 (81%)	57 ± 12	NA	28.6%	NA	OGTT, HbA1c	32 ± 52
Walker et al. (62)	Scotland	Prospective cohort	AP	1,748 (49%)	NA	NA	13.3%	NA	Diagnostic codes, Medication prescriptions	73 (62–84)
Wu et al. (58)	China	Prospective cohort	AP	59 (56%)	59 ± 14	NA	NA	30.5%	FPG, HbA1c	42^**^ (12-72)^||^
Wundsam et al. (80)^‡^	Austria	Retrospective cohort	AP	302 (59%)	60 ± 18	NA	3.3%	NA	NA	NA
Yu et al. (31)	China	Retrospective cohort	AP	361 (56%)	49 ± 13	NA	NA	41.6%	FPG, OGTT	24 ± 24
Yuan et al. (27)	China	Retrospective cohort	AP	310 (60%)	52 (41–63)	11.0%	11.3%	22.3%	FPG	36 (22–53)
Zhang et al. (81)^† ‡^	China	Retrospective cohort	AP	946 (NA)	NA	NA	7.0%	NA	NA	0–48^||^
Zhang et al. (49)	China	Retrospective cohort	AP	820 (61.3%)	50 (38–63)	NA	8.3%	NA	Diagnostic codes	3–57^||^

NA, Not available; AP, Acute pancreatitis; ANP, Acute necrotizing pancreatitis; CP, Chronic pancreatitis; SAP, Severe acute pancreatitis; MSAP, Moderately severe acute pancreatitis; MAP, Mild acute pancreatitis; DM, Diabetes mellitus; PD, Prediabetes; FPG, Fasting plasma glucose; OGTT, Oral glucose tolerance test; HbA1c, Hemoglobin A1c; G, Group; PEI, Pancreatic exocrine insufficiency; BMI, Body mass index; ^*^Data reported as mean with standard deviation or median with interquartile range, unless otherwise specified; ^†^Conference abstract; ^‡^Study not included in the meta-analyses; ^§^Median; ^¶^SD; ^||^Range; ^**^Mean; ^††^Standard error of the mean; ^‡‡^Standard error; and ^§§^Predetermined follow-up time.

### 3.3 Synthesis of results

**Figure 2:**
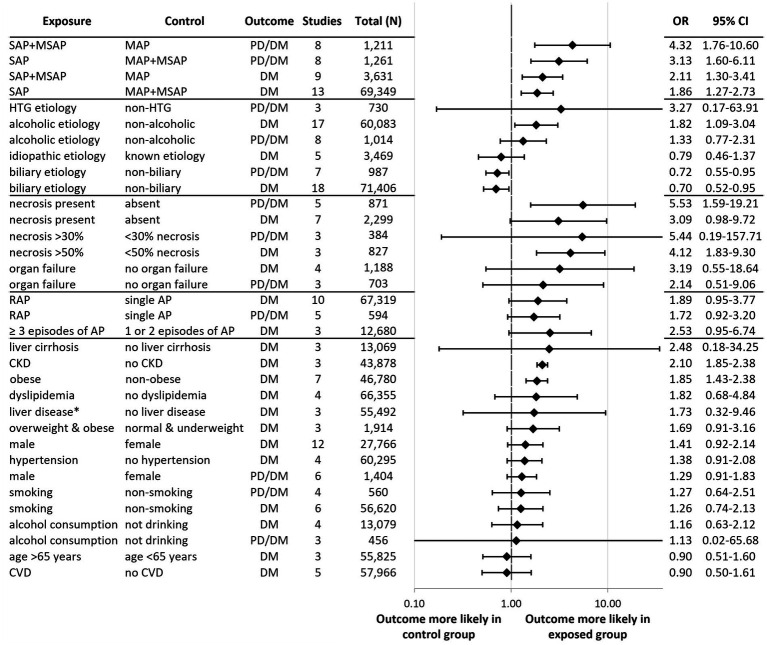
Aggregated forest plot summarizing our results for the 34 meta-analyses. Each row shows the pooled odds ratio for a risk factor and outcome pair. An odds ratio over 1.0 indicates that the given outcome (diabetes or PD/DM) is more likely to occur in the exposed group compared to the control group. Statistical significance is achieved if the line of null effect does not fall into the confidence interval. Black squares represent the pooled odds ratios and the lines represent the confidence intervals. PD, Prediabetes; DM, Diabetes mellitus; OR, Odds ratio; CI, Confidence interval; AP, Acute pancreatitis; SAP, Severe acute pancreatitis; MSAP, Moderately severe acute pancreatitis; MAP, Mild acute pancreatitis; HTG, Hypertriglyceridemic; RAP, Recurrent acute pancreatitis; CKD, Chronic kidney disease; and CVD, Cardiovascular disease. ^*^Liver disease other than liver cirrhosis.

Our findings of the 34 meta-analyses are summarized in an aggregated forest plot, which shows the pooled OR for each risk factor and outcome pair (Figure 2). In addition, per risk factor groups we present the original forest plots or more detailed aggregated forest plots. All other individual plots can be found in the Supplementary material.

#### 3.3.1 AP severity and complications

**Figure 3:**
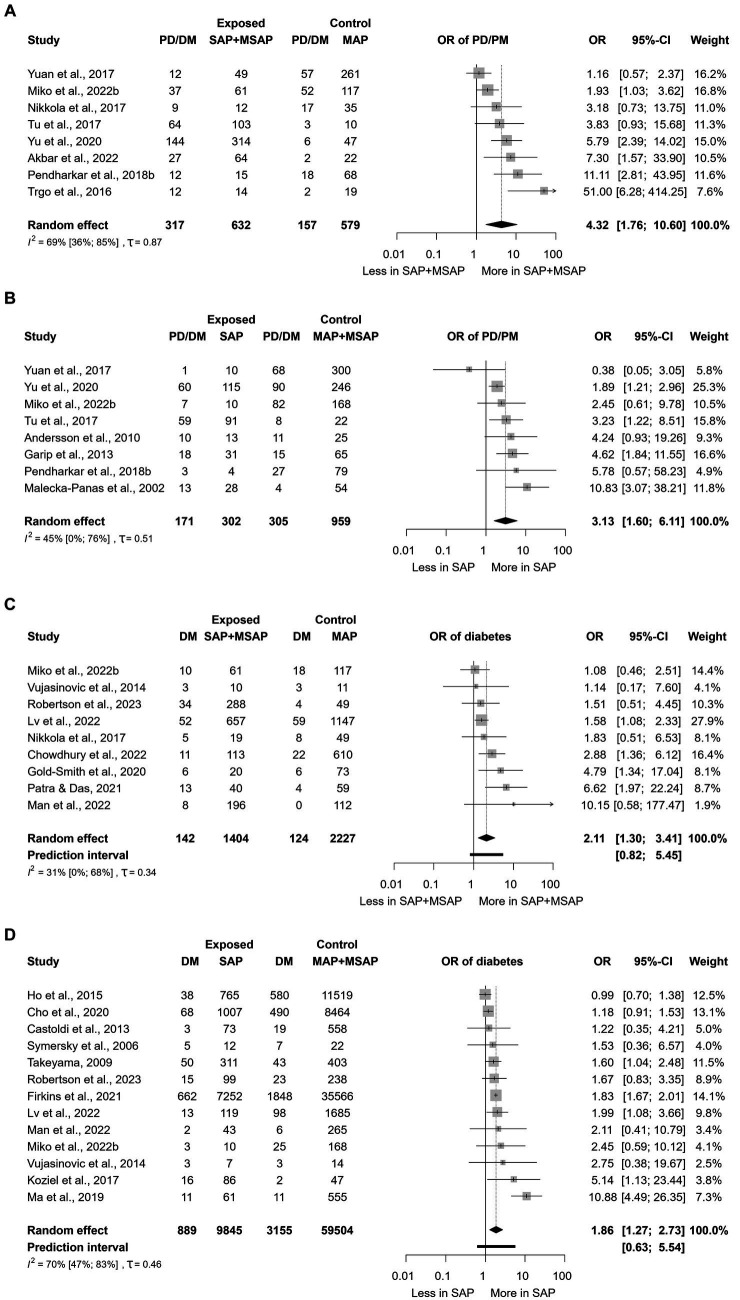
The association between severity grades of acute pancreatitis (AP) and subsequent development of prediabetes and diabetes. **(A)** Severe or moderately severe AP vs. mild AP in relation to new-onset prediabetes and diabetes. **(B)** Severe AP vs. mild or moderately severe AP in relation to new-onset prediabetes and diabetes. **(C)** Severe or moderately severe AP vs. mild AP and new-onset diabetes. **(D)** Severe AP vs. mild or moderately severe AP and new-onset diabetes. AP, Acute pancreatitis; OR, Odds ratio; CI, Confidence interval; PD, Prediabetes; DM, Diabetes mellitus; SAP, Severe acute pancreatitis; MSAP, Moderately severe acute pancreatitis; MAP, Mild acute pancreatitis; and vs., versus.

Having SAP or moderately severe AP was associated with a significantly greater odds of developing PD/DM [OR: 4.32; CI: 1.76–10.60; Figure 3A; (27–34)] and DM [OR: 2.11; CI: 1.30–3.41; Figure 3C; (28, 29, 35–41)] compared to mild disease. SAP was associated with significantly increased odds of developing PD/DM [OR: 3.13; CI: 1.60–6.11; Figure 3B; (18, 22, 23, 27, 28, 30, 31, 33)] and DM [OR: 1.86; CI: 1.27–2.73; Figure 3D; (19–21, 24, 28, 35–37, 41–45)] compared to mild-or-moderate disease.

**Figure 4:**
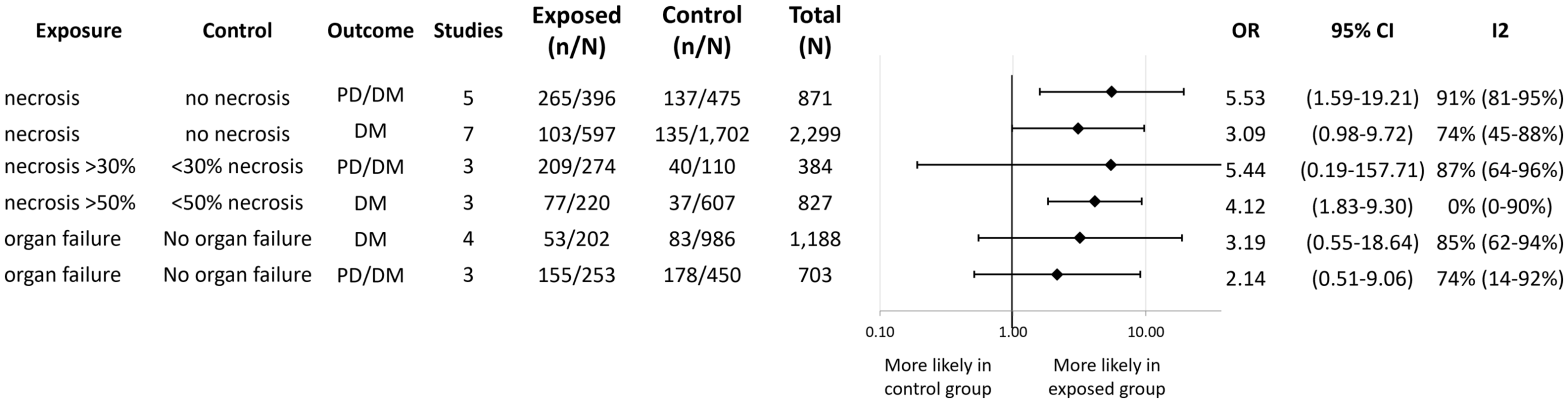
Aggregated forest plot showing the pooled odds ratios for various complications of acute pancreatitis and subsequent diabetes and prediabetes development. PD, Prediabetes; DM, Diabetes mellitus; OR, Odds ratio; and CI, Confidence interval.

We found a significantly greater odds of developing PD/DM with necrotizing AP [OR: 5.53; CI: 1.59–19.21; (22, 31, 46–48)] and a statistically non-significant tendency with DM [OR: 3.09; CI: 0.98–9.72; (24, 30, 36, 40, 41, 49, 50)] compared to non-necrotizing AP (Figure 4). Sensitivity analysis revealed that leaving out Takeyama (24) from the analysis would lead to a statistically significant OR (4.17; CI: 2.08–8.37) of developing DM in AP patients who had necrosis compared to its absence (Supplementary Figure S28). In this study, the data collection of index AP episode—and thus the evaluation of necrosis—occurred in 1987, which was 24 years earlier than any other study included in the analysis. Notably, computer tomography imaging has improved significantly in that time (51).

A limited number of studies allowed for the analysis of the extent of pancreatic necrosis (Figure 4). Necrosis affecting over 50% of the pancreatic tissue was associated with a significantly higher odds of developing DM [OR: 4.12; CI: 1.83–9.30; (30, 36, 41)] compared to smaller proportions affected. We also observed a statistically non-significant tendency for developing PD/DM in patients whose pancreas was at least 30% necrotic [OR: 5.44; CI: 0.19–157.71; (30–32)].

Similarly, only a statistically non-significant tendency could be observed in case of any organ failure (regardless of organ and duration of impairment) and DM [OR: 3.19; CI: 0.55–18.64; (36, 40, 45, 52)] or PD/DM [OR: 2.14; CI: 0.51–9.06; (31, 32, 46); Figure 4].

#### 3.3.2 AP etiology and recurrent AP

**Figure 5:**
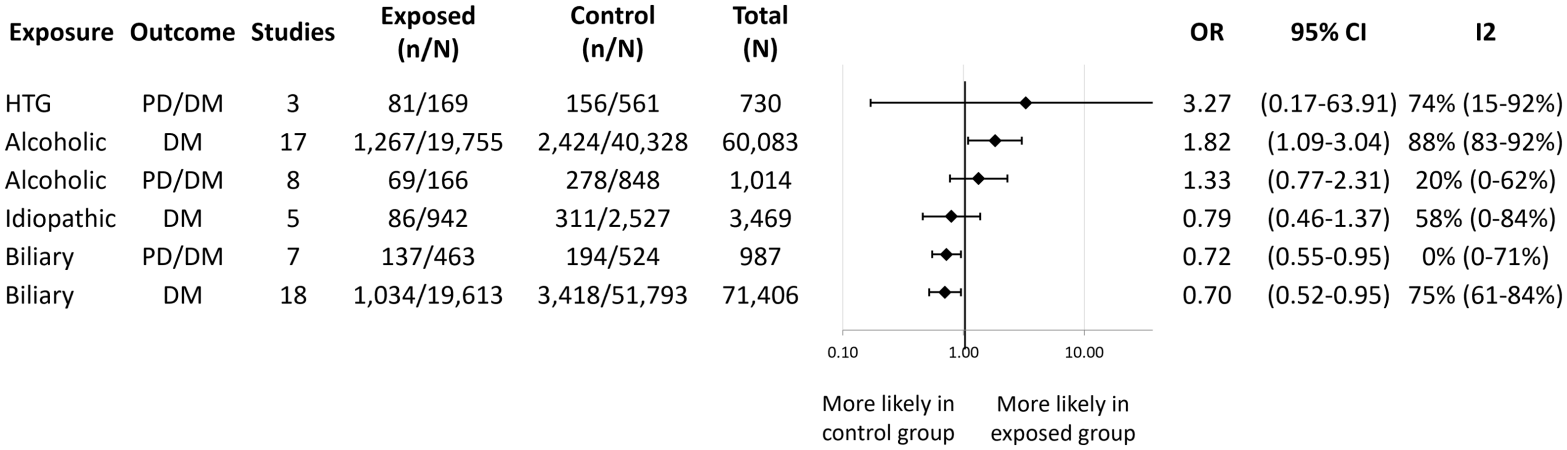
Aggregated forest plot showing the pooled odds ratios for different etiologies of acute pancreatitis and new-onset diabetes alone or in combination with prediabetes. Etiologies listed in the exposure column are compared to all other etiologies to provide an odds ratio for the outcome of interest. PD, Prediabetes; DM, Diabetes mellitus; HTG, Hypertriglyceridemic; OR, Odds ratio; and CI, Confidence interval.

We conducted quantitative syntheses assessing the risk of PD/DM after alcoholic, biliary, and hypertriglyceridemia-induced AP, and the risk of DM after alcoholic, biliary, and idiopathic AP (Figure 5). We found that alcoholic AP patients had a higher odds of developing DM [OR: 1.82; CI: 1.09–3.04; *I*^2^ = 88%; (18, 20, 24, 35–40, 43, 45, 50, 53–57)] compared to patients with non-alcoholic AP. Moreover, after conducting a subgroup analysis based on follow-up time, we found reduced statistical heterogeneity (*I*^2^ = 57%) as well as a possible increasing effect over time (Supplementary Figure S6). While not reaching statistical significance, we observed a tendency of increased risk of new-onset PD/DM following alcoholic [OR: 1.33; CI: 0.77–2.31; (23, 27, 31, 33, 53, 57–59)] and hypertriglyceridemic AP [OR: 3.27; CI: 0.17–63.91; (27, 31, 58)] as well. Biliary etiology was associated with a significantly lower odds of developing DM [OR: 0.70; CI: 0.52–0.95; (18, 20, 24, 35–38, 41–43, 45, 50, 54, 55, 57, 60–62)] and PD/DM [OR: 0.72; CI: 0.55–0.95; (23, 27, 31, 33, 47, 57, 58)] compared to other etiologies. A statistically non-significant reducing trend could be observed for idiopathic AP and DM development [OR: 0.79; CI: 0.46–1.37; (24, 37, 45, 54, 55)].

**Figure 6:**
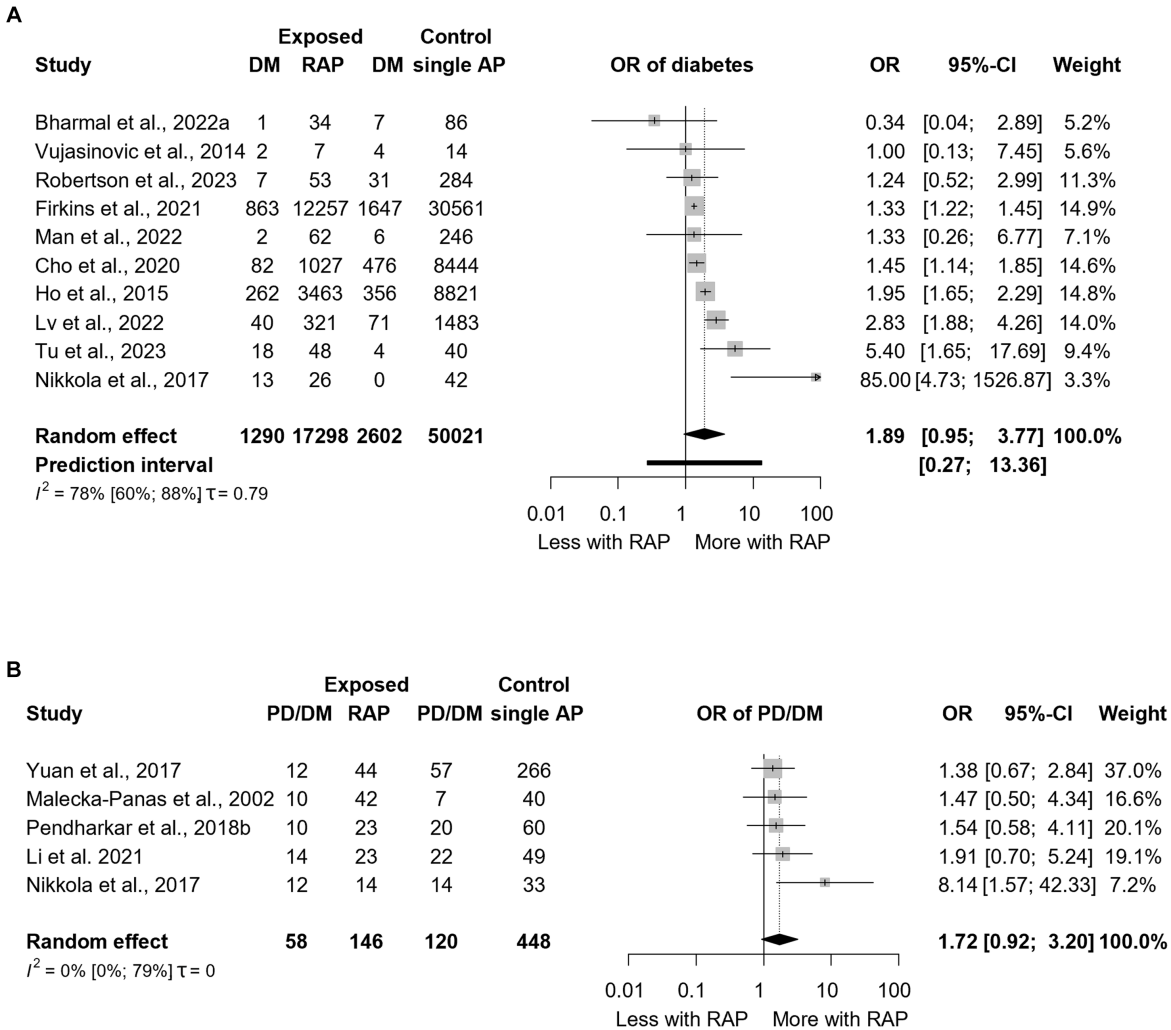
The association between recurrent acute pancreatitis and subsequent development of diabetes **(A)** and prediabetes or diabetes **(B)**. PD, Prediabetes; DM, Diabetes mellitus; OR, Odds ratio; CI, Confidence interval; AP, Acute pancreatitis; and RAP, Recurrent acute pancreatitis.

We observed a near statistically significant increased odds of DM [OR: 1.89; CI: 0.95–3.77; Figure 6A; (4, 20, 29, 35–37, 41–43, 50)] and PD/DM [OR: 1.72; CI: 0.92–3.20; Figure 6B; (23, 27, 29, 33, 47)] recurrent acute pancreatitis (RAP) compared to a single AP episode. Subgroup analysis for follow-up length found no effect of time; however, few studies made up each subgroup. Some studies explored the effect of different numbers of AP episodes. Three or more episodes of AP were associated with a near statistically significant increased odds of DM [OR: 2.53; CI: 0.95–6.74; (4, 20, 41)] compared to having one or two AP episodes (Supplementary Figure S10).

#### 3.3.3 Demographic factors and comorbidities

**Figure 7:**
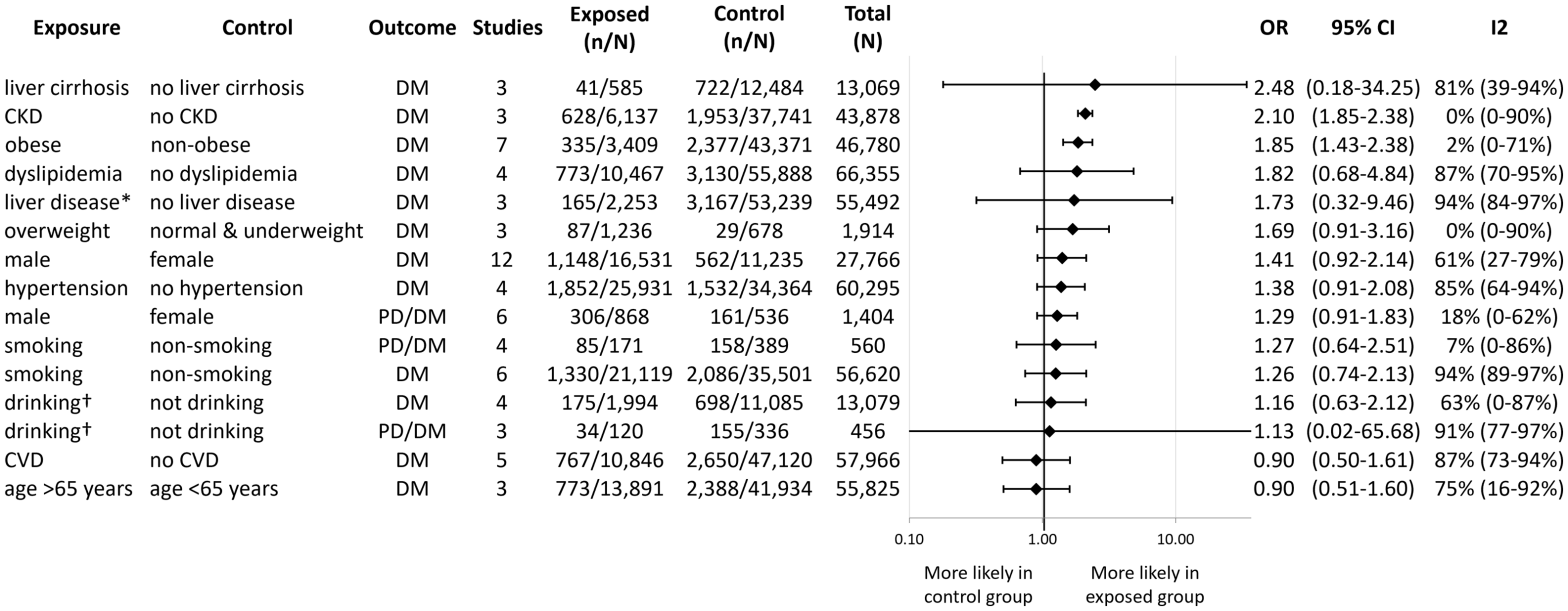
Aggregated forest plot showing the pooled odds ratios for various comorbidities, demographic factors, and new-onset diabetes alone or in combination with prediabetes. PD, Prediabetes; DM, Diabetes mellitus; OR, Odds ratio; CI, Confidence interval; CKD, Chronic kidney disease; and CVD, Cardiovascular disease. ^*^Liver diseases other than liver cirrhosis. ^†^Drinking refers to alcohol consumption.

Figure 7 displays the pooled OR for the remainder of the prognostic factors that were reported on by a sufficient number of included studies in a comparable manner for quantitative synthesis (see Supplementary Figures S11–S22 for individual forest plots). We found that obesity (29, 39, 41, 43, 49, 50, 62) and chronic kidney disease (36, 38, 43) were associated with a significantly higher odds of developing DM (OR: 1.85; CI: 1.43–2.38 and OR: 2.10; CI: 1.85–2.38, respectively). We observed a statistically non-significant tendency of increased odds of developing DM with liver cirrhosis (20, 38, 63), other liver disease (37, 43, 64), dyslipidemia (20, 37, 42, 43), and being overweight or obese (37, 41, 50). We found no association between new-onset DM and hypertension (20, 36, 37, 43), cardiovascular disease (20, 36–38, 43), or age (20, 38, 43). Smoking (29, 31, 36–38, 43, 64–66), alcohol consumption (29, 31, 36, 37, 64, 67), and male sex (20, 27, 31–33, 35–38, 41, 42, 47, 50, 61, 62, 68–70) were not associated with either new-onset DM or PD/DM.

#### 3.3.4 Additional risk factors and outcomes

There were 55 additional prognostic factors investigated by the included studies that could not be meta-analyzed due to an insufficient number of reports or heterogeneity. See Supplementary Table S5 for the qualitative analysis, which includes the 11 eligible studies that could not be meta-analyzed (71–81).

### 3.4 Evaluation of bias and heterogeneity

Overall, the proportion of the high risk of bias studies was notable (32–44%) for all three outcome factors (Supplementary Figures S23–S25). This was primarily due to a lack of reporting on study attrition and suboptimal definitions of outcome measurements.

High heterogeneity was noted in several of our analyses. Subgroup analysis for follow-up length significantly reduced heterogeneity only for new-onset DM in relation to alcoholic etiology. For the other prognostic factors, heterogeneity remained high even after accounting for follow-up time.

Of the 34 risk factor and outcome pairs that could be meta-analyzed, sensitivity analysis was feasible in the case of 14 analyses (Supplementary Figures S26–S34). Leave-one-out analysis identified one study (24), whose omission would make a significant difference, which we reported in paragraph 3.3.1.

Publication bias assessment was limited to six meta-analyses on new-onset DM: severe AP, moderately severe and severe AP, alcoholic and biliary etiology, recurrent AP, and male sex (Supplementary Figures S35–S40). Possible small study publication bias was detected in the case of alcoholic etiology in relation to DM development based on Egger’s test and visual inspection of the funnel plot.

## 4 Discussion

This is the first systematic review and meta-analysis of risk factors for developing new-onset PD and DM after AP that pooled direct, within-study comparisons. We found that severe AP, moderately severe AP, and necrosis are associated with a greater risk of developing DM and PD/DM. We also observed a significant association with alcoholic etiology, obesity, chronic kidney disease, and new-onset DM, whereas biliary etiology was associated with a lower risk of developing DM and PD/DM compared to other etiologies. Additionally, we observed a tendency for increased risk of developing DM or PD/DM with hypertriglyceridemic AP, organ failure, RAP, and comorbidities of liver disease or dyslipidemia.

### 4.1 Severity and local complications

Past meta-analyses applying indirect comparisons found conflicting results regarding the association of AP severity and new-onset PD or DM (7, 8). Our analysis of direct, within-study comparisons confirms a positive relationship between SAP, moderately severe AP, and new-onset DM. Classification of AP severity is based on the development of local complications (such as necrosis) and organ failure (1). Beta cell death secondary to local complications of AP is believed to be one of the possible mechanisms behind the ensuing DM (82). Our meta-analysis supports this hypothesis as necrosis was associated with significantly greater risk of developing DM and PD/DM. Moreover, patients with local complications might require interventions such as pancreatic debridement, lavage, drainage, necrosectomy, and partial pancreatectomy, during which further pancreatic tissue is lost (83).

Nevertheless, cell death is only one aspect of the complex pathomechanism of post-AP DM. It was proposed that the inflammation accompanying AP stimulates endogenous beta-cell proteins to undergo post-translational modifications (84). Such modified proteins could trigger autoimmune processes as seen in type 1 diabetes (85), which could explain the earlier and greater need for insulin therapy seen with post-pancreatitis DM compared to type 2 DM (12). The level of inflammatory cytokines correlate well with persistent organ failure, which is the hallmark of SAP (86). Our study found an increase in the odds of developing DM and PD/DM after AP with organ failure, albeit not-statistically significant. It is noteworthy that none of the included studies specified the duration of the organ failure, and few mentioned the affected organs or the number of organs affected.

We found a more pronounced association with severity in the case of PD/DM than with DM suggesting an even more substantial influence of AP severity on the development of PD. Moreover, comparing severe and moderate AP as one group vs. mild yielded a higher odds ratio than the comparison of SAP to moderate and mild AP as one group. This could imply that progresses from mild to moderate AP severity has a greater impact on PD and DM development compared to the step from moderate to severe disease progression.

### 4.2 Etiology

We found that alcoholic AP was associated with an increased risk of developing DM. Alcohol has a toxic effect on the pancreas. Its metabolites elicit sustained intracellular calcium overload, which disrupts beta-cell functioning and insulin secretion while also leading to oxidative stress (87), to which beta-cells are especially vulnerable due to their low antioxidation capacity (88).

The most likely explanation for the tendency seen with hypertriglyceridemic etiology is that hypertriglyceridemia itself is associated with DM (89). The two conditions often coexist in metabolic syndrome. Analysis of the Hungarian Study Group’s registry data shows that 69% of the non-diabetic hypertriglyceridemic AP patients present with at least two factors of the metabolic syndrome on admission and they are at an increased risk of developing post-AP DM (90). Therefore, the development of DM might be a natural progression of the disease, possibly quickened by the AP episode.

Acute pancreatitis tends to be more severe if caused by excessive alcohol consumption or hypertriglyceridemia (91) and if metabolic syndrome is present (90). Toxic factors (e.g., alcohol and fatty acids) play a role in the development and severity of pancreatitis when they accumulate (92). This aligns with the multiple hits theory of AP severity documented for smoking, drinking (93), obesity, hypertension, and hyperlipidemia (90). The risk factors we identified—local complications, severity, alcoholic, and hypertriglyceridemic AP—often coexist (91, 94, 95). Suggesting that the development of post-AP DM might work on a similar multiple hits theory basis.

Both alcoholic and hypertriglyceridemic etiologies are linked to poor dietary habits that are difficult to change and the ongoing exposure conveys a high risk for RAP, progression of the disease, and development of complications (96). On the contrary, the recurrence of biliary AP is often prevented by cholecystectomy after the index episode (64). Without identifying a treatable or preventable etiology, there is a risk for RAP. However, we found no association between idiopathic AP and DM development, possibly due to the control group—containing alcoholic and hypertriglyceridemic etiology—demonstrating a positive association with DM.

### 4.3 Recurrence

Recurrent acute pancreatitis conveys repeated pancreatic inflammation and cellular insult or loss, leading to an assumed association with developing pancreatic endocrine dysfunction (96). Our analysis found a tendency of increased odds for new-onset DM and PD/DM with RAP, which neared statistical significance. It should be pointed out that there was considerable heterogeneity in the study designs. Some studies excluded patients presenting with RAP at the index AP episode while others included them. Importantly, those who had RAP and developed DM by the index AP episode were excluded from the analysis based on the premise of pre-existing DM. Moreover, 60% of the analyzed studies had a relatively short follow-up of less than 3 years. Finally, different distributions of the etiological factors among the included studies might influence the observed association between RAP and DM or PD/DM, as alcoholic and hypertriglyceridemic APs are associated with a greater risk of RAP (91). All four factors could influence the true relationship between disease recurrence and PD/DM development.

### 4.4 Other factors

Our study found that obesity was associated with a significantly greater risk of new-onset DM. Some of the other risk factors we identified for new-onset DM after AP (hypertriglyceridemic AP, AP-related complications, and SAP) tend to occur more frequently in obese individuals (90, 97). Moreover, excess weight is a known independent risk factor for type 2 DM. Therefore, it could be a natural progression of the disease or AP might even trigger DM in genetically or metabolically predisposed patients (7). At present, there is still a lack of consensus on differentiating type 2 DM from post-AP DM in patients who had an AP episode (7, 98). Most studies define post-AP DM as new-onset of hyperglycemia (using the standard cutoff values for DM as per the World Health Organization or American Diabetes Association recommendations) following an AP episode (12, 13, 99). The prospective, multi-center DREAM study (Diabetes RElated to Acute Pancreatitis and Its Mechanisms) was recently designed to characterize the DM phenotypes after AP and their pathomechanism (100, 101).

We found no association between sex, smoking, alcohol consumption, and DM or PD/DM. The lack of association with alcohol consumption is paradoxical in light of the increased risk of DM with AP of alcoholic etiology. However, the included studies mostly compared alcohol consumption to not-drinking, not taking into account the amount and duration of alcohol consumption. Studies with follow-up length over 4 years were more likely to favor an association between alcohol consumption and new-onset DM or PD/DM (29, 64, 67) compared to shorter studies (31, 36, 37). Also, the analysis included only four and three studies for DM and PD/DM, respectively, with two of the studies containing unusually low proportions of alcoholic etiology (4 and 14%) and three studies including only patients with AP of alcoholic etiology.

Additionally, we observed a clinically relevant odds ratio for post-AP DM with liver disease and dyslipidemia. We believe that statistical significance was not achieved due to the low number of studies investigating these risk factors and their heterogeneous nature. In our analysis, chronic kidney disease was associated with a significantly higher risk of post-AP DM. It is notable that the analysis was based on three studies, of which Firkins et al. (43) accounted for 99.4% of the pooled results due to the large sample size. This is a retrospective nationwide database analysis, where only patients with a second hospital admission within one calendar year were included. Patients with chronic kidney disease are admitted more frequently to hospitals (102); thus, they were likely over-represented in the study by Firkins et al. (43).

### 4.5 Follow-up after AP

Timely translation of scientific data to clinical practice has crucial importance in healthcare (103, 104). Long-term complications of AP (exocrine and endocrine insufficiency) were documented as early as 1941 (105); nonetheless, the Chinese guideline in 2021 was the first to recommend follow-up visits after AP (59). They recommend that all AP patients should be monitored after rehabilitation, however, for different lengths of time depending on severity. They rated the strength of recommendation and supporting evidence weak.

While all AP patients should be followed up for the development of long-term complications after AP, financial and human resources are often limited in healthcare. Our study highlights the sub-populations of AP patients who are at a higher risk for developing PD or DM. Therefore, more frequent follow-ups of these patients increase the likelihood of preventing and reducing post-AP diabetes-related morbidity, mortality, and healthcare costs.

### 4.6 Strengths and limitations

Due to the broad search strategy and lack of constraints on the results, this is the first comprehensive systematic analysis of potential risk factors for new-onset PD/DM following AP, with the largest number of included studies (50 in total) covering 76,797 participants in the meta-analysis. Our study was based on direct, within-study comparisons; therefore, it is more representative of the true effect of risk factors compared to previous meta-analyses (7, 8). Due to the inclusive nature of our research, there was substantial heterogeneity between the studies, which we attempted to reduce by performing separate analyses for PD/DM and DM and conducting subgroup analysis for follow-up length. Almost a third of the meta-analyses were based on three studies. For these risk factor and outcome pairs, conclusions should be cautiously handled.

### 4.7 Implication for practice

All patients require medical follow-up for endocrine and exocrine insufficiency after AP. Our results show that patients who have suffered severe or moderately severe AP, alcoholic or hypertriglyceridemic AP, develop pancreatic necrosis or organ failure, had multiple AP episodes, are obese or have pre-existing chronic kidney disease, liver disease or dyslipidemia are at a greater risk for developing PD or DM. Therefore, closer monitoring is warranted in these high-risk groups.

### 4.8 Implication for research

Further long-term follow-up studies of AP patients are needed to observe morbidity and mortality following single and multiple AP episodes as well. High-quality well-controlled observational studies with long follow-up duration are needed to establish an evidence-based follow-up schedule after AP to help identify patients early in a prediabetic state, where interventions could still prevent DM. Future studies should also explore interventions for preventing post-pancreatitis DM. In 2022 the Hungarian Pancreatic Study Group launched two longitudinal randomized controlled trials on dietary intervention (106) and smoking and alcohol cessation following hospitalization for AP (107).

### 4.9 Conclusion

We found that AP severity, alcoholic and hypertriglyceridemic etiologies, pancreatic necrosis, organ failure, RAP and comorbidities of obesity, chronic kidney disease, liver disease, and dyslipidemia are associated with a higher risk of developing PD or DM following AP. Glucose homeostasis should be regularly monitored in high-risk populations after hospital discharge. Further research is needed to establish an appropriate follow-up schedule and interventions for preventing DM after AP.

## Data availability statement

The original contributions presented in the study are included in the article/Supplementary material, further inquiries can be directed to the corresponding author.

## Author contributions

OZ: Conceptualization, Writing – review & editing, Data curation, Investigation, Methodology, Project administration, Visualization, Writing – original draft. SB: Conceptualization, Methodology, Project administration, Visualization, Writing – review & editing. AK: Investigation, Writing – review & editing. DD: Investigation, Writing – review & editing, Visualization. LH: Visualization, Investigation, Writing – review & editing. BB: Investigation, Writing - review & editing. DV: Data curation, Visualization, Conceptualization, Writing – review & editing, Formal analysis, Methodology. NH: Conceptualization, Writing – review & editing, Methodology. BE: Writing – review & editing, Conceptualization, Methodology. BT: Writing – review & editing, Methodology, Project administration, Visualization. MJ: Writing – review & editing, Conceptualization, Methodology, Supervision. PH: Conceptualization, Funding acquisition, Supervision, Writing – review & editing, Methodology.

## Glossary

**Table:** 

ANP	Acute necrotizing pancreatitis
AP	acute pancreatitis
BMI	Body mass index
CENTRAL	Cochrane central register of controlled trials
CI	95% Confidence interval
CKD	Chronic kidney disease
CP	Chronic pancreatitis
CVD	Cardiovascular disease
DM	Diabetes mellitus
DREAM	Diabetes RElated to Acute Pancreatitis and Its Mechanisms
FPG	Fasting plasma glucose
G	Group
HbA1c	Hemoglobin A1c
HTG	Hypertriglyceridemic
MAP	Mild acute pancreatitis
MSAP	Moderately severe acute pancreatitis
NA	Not available
OGTT	Oral glucose tolerance test
OR	Odds ratio
PD	Prediabetes
PECOTS	Population, exposure, comparator, outcome, timing, study design
PEI	Pancreatic exocrine insufficiency
PRISMA	Preferred Reporting Items for Systematic Reviews and Meta-Analyses
PROSPERO	International Prospective Register of Systematic Reviews
QUIPS	Quality In Prognosis Studies
RAP	Recurrent acute pancreatitis
SAP	Severe acute pancreatitis
