# Supplementary material for: Risk factors for diabetes mellitus after acute pancreatitis: a systematic review and meta-analysis
Source: Front Med (Lausanne). 2024 Jan 9;10:1257222. doi: 10.3389/fmed.2023.1257222 (PMC10803425; doi:10.3389/fmed.2023.1257222)
Supplement: Supplementary file 1 [file Data_Sheet_1.pdf]

Supplementary material

Supplement to: **Risk factors for diabetes mellitus after acute pancreatitis: a systematic review and meta-analysis**

Table of Contents

Table of Contents 2
METHODS 4
Table S.1 – PRISMA checklist 4
Table S.2 – Search key, selection process and data collection process 7
Table S.3 – List of prognostic factors 8
Table S.4 – Data synthesis and statistical analysis 9
RESULTS: Forest plots of risk factor & outcome pairs 11
Figure S.1 – Necrosis & risk of A) diabetes and B) prediabetes/ diabetes 11
Figure S.2 – Necrosis >30% of pancreas & risk of prediabetes/ diabetes 11
Figure S.3 – Necrosis >50% of pancreas & risk of diabetes 12
Figure S.4 – Organ failure & risk of A) diabetes and B) prediabetes/ diabetes 12
Figure S.5 – AP etiology: hypertriglyceridemic & risk of prediabetes/ diabetes 13
Figure S.6 – AP etiology: alcoholic & risk of diabetes 13
Figure S.7 – AP etiology: alcoholic & risk of prediabetes/diabetes 14
Figure S.8 – AP etiology: biliary & risk of A) diabetes and B) prediabetes/ diabetes 14
Figure S.9 – AP etiology: idiopathic & risk of diabetes 15
Figure S.10 – Number of AP episodes ≥3 & risk of diabetes 15
Figure S.11 – Comorbidity: obesity & risk of diabetes 16
Figure S.12 – Comorbidity: overweight and obese & risk of diabetes 16
Figure S.13 – Comorbidity: dyslipidemia & risk of diabetes 16
Figure S.14 – Comorbidity: liver cirrhosis & risk of diabetes 17
Figure S.15 – Comorbidity: other liver diseases & risk of diabetes 17
Figure S.16 – Comorbidity: chronic kidney disease & risk of diabetes 17
Figure S.17 – Comorbidity: hypertension & risk of diabetes 18
Figure S.18 – Comorbidity: cardiovascular disease & risk of diabetes 18
Figure S.19 – Alcohol consumption & risk of A) diabetes B) prediabetes/diabetes 18
Figure S.20 – Smoking & risk of A) diabetes B) prediabetes/diabetes 19
Figure S.21 – Sex & risk of A) diabetes B) prediabetes/diabetes 20
Figure S.22 – Age & risk of diabetes 21
RESULTS: Qualitative analysis 22
Table S.5 – Qualitative summary 22
RESULTS: Risk of bias assessment 26
Figure S.23 – Risk of bias assessment: prediabetes 26

Figure S.24 – Risk of bias assessment: diabetes 27
Figure S.25 – Risk of bias assessment: prediabetes/diabetes 29
RESULTS: Sensitivity analysis 31
Figure S.26 – Sensitivity analysis: severe AP 31
Figure S.27 – Sensitivity analysis: severe or moderate AP 31
Figure S.28 – Sensitivity analysis: necrosis 32
Figure S.29 – Sensitivity analysis: alcoholic AP 32
Figure S.30 – Sensitivity analysis: biliary AP 33
Figure S.31 – Sensitivity analysis: recurrent AP 34
Figure S.32 – Sensitivity analysis: smoking 34
Figure S.33 – Sensitivity analysis: obesity 34
Figure S.34 – Sensitivity analysis: male sex 35
RESULTS: Publication bias 36
Figure S.35 – Publication bias assessment: severe AP 36
Figure S.36 – Publication bias assessment: severe or moderate AP 36
Figure S.37 – Publication bias assessment: alcoholic AP 37
Figure S.38 – Publication bias assessment: biliary AP 37
Figure S.39 – Publication bias assessment: recurrent AP 38
Figure S.40 – Publication bias assessment: male sex 38
REFERENCES 39

METHODS

Table S.1 – PRISMA checklist

Section and Topic	Item #	Checklist item	Location where item is reported
**TITLE**			
Title	1	Identify the report as a systematic review.	p.1
**ABSTRACT**			
Abstract	2	See the PRISMA 2020 for Abstracts checklist.	p.1
**INTRODUCTION**			
Rationale	3	Describe the rationale for the review in the context of existing knowledge.	p.2
Objectives	4	Provide an explicit statement of the objective(s) or question(s) the review addresses.	p.2
**METHODS**			
Eligibility criteria	5	Specify the inclusion and exclusion criteria for the review and how studies were grouped for the syntheses.	p.2
Information sources	6	Specify all databases, registers, websites, organizations, reference lists and other sources searched or consulted to identify studies. Specify the date when each source was last searched or consulted.	p.2
Search strategy	7	Present the full search strategies for all databases, registers and websites, including any filters and limits used.	p.2, Suppl p.7
Selection process	8	Specify the methods used to decide whether a study met the inclusion criteria of the review, including how many reviewers screened each record and each report retrieved, whether they worked independently, and if applicable, details of automation tools used in the process.	p.2, Suppl p.7
Data collection process	9	Specify the methods used to collect data from reports, including how many reviewers collected data from each report, whether they worked independently, any processes for obtaining or confirming data from study investigators, and if applicable, details of automation tools used in the process.	p.3, Suppl p.7
Data items	10a	List and define all outcomes for which data were sought. Specify whether all results that were compatible with each outcome domain in each study were sought (e.g. for all measures, time points, analyses), and if not, the methods used to decide which results to collect.	p.2-3
	10b	List and define all other variables for which data were sought (e.g. participant and intervention characteristics, funding sources). Describe any assumptions made about any missing or unclear information.	p.3, Suppl p.8-9
Study risk of bias	11	Specify the methods used to assess risk of bias in the included studies, including details of the tool(s) used, how many	p.3

Section and Topic	Item #	Checklist item	Location where item is reported
assessment		reviewers assessed each study and whether they worked independently, and if applicable, details of automation tools used in the process.	
Effect measures	12	Specify for each outcome the effect measure(s) (e.g. risk ratio, mean difference) used in the synthesis or presentation of results.	p.3, Suppl p.9
Synthesis methods	13a	Describe the processes used to decide which studies were eligible for each synthesis (e.g. tabulating the study intervention characteristics and comparing against the planned groups for each synthesis (item #5)).	Suppl p.9
	13b	Describe any methods required to prepare the data for presentation or synthesis, such as handling of missing summary statistics, or data conversions.	Suppl p.9
	13c	Describe any methods used to tabulate or visually display results of individual studies and syntheses.	Suppl p.9
	13d	Describe any methods used to synthesize results and provide a rationale for the choice(s). If meta-analysis was performed, describe the model(s), method(s) to identify the presence and extent of statistical heterogeneity, and software package(s) used.	Suppl p.9
	13e	Describe any methods used to explore possible causes of heterogeneity among study results (e.g. subgroup analysis, meta-regression).	Suppl p.10
	13f	Describe any sensitivity analyses conducted to assess robustness of the synthesized results.	Suppl p.9
Reporting bias assessment	14	Describe any methods used to assess risk of bias due to missing results in a synthesis (arising from reporting biases).	p.3 Suppl p.9-10
Certainty assessment	15	Describe any methods used to assess certainty (or confidence) in the body of evidence for an outcome.	NA
**RESULTS**			
Study selection	16a	Describe the results of the search and selection process, from the number of records identified in the search to the number of studies included in the review, ideally using a flow diagram.	p.3, Fig.1
	16b	Cite studies that might appear to meet the inclusion criteria, but which were excluded, and explain why they were excluded.	NA
Study characteristics	17	Cite each included study and present its characteristics.	p.5-8
Risk of bias in studies	18	Present assessments of risk of bias for each included study.	Suppl p.26-30
Results of individual studies	19	For all outcomes, present, for each study: (a) summary statistics for each group (where appropriate) and (b) an effect estimate and its precision (e.g. confidence/credible interval), ideally using structured tables or plots.	NA
Results of	20a	For each synthesis, briefly summarise the characteristics and risk of bias among contributing studies.	p.3-4, 8

Section and Topic	Item #	Checklist item	Location where item is reported
syntheses	20b	Present results of all statistical syntheses conducted. If meta-analysis was done, present for each the summary estimate and its precision (e.g. confidence/credible interval) and measures of statistical heterogeneity. If comparing groups, describe the direction of the effect.	p. 3-4, 8, Suppl p.11-21
	20c	Present results of all investigations of possible causes of heterogeneity among study results.	p.8, Suppl p.13
	20d	Present results of all sensitivity analyses conducted to assess the robustness of the synthesized results.	p.8, Suppl p.31-33
Reporting biases	21	Present assessments of risk of bias due to missing results (arising from reporting biases) for each synthesis assessed.	p.8, Suppl p.36-38
Certainty of evidence	22	Present assessments of certainty (or confidence) in the body of evidence for each outcome assessed.	NA
**DISCUSSION**			
Discussion	23a	Provide a general interpretation of the results in the context of other evidence.	p.8-13
	23b	Discuss any limitations of the evidence included in the review.	p.13
	23c	Discuss any limitations of the review processes used.	p.13
	23d	Discuss implications of the results for practice, policy, and future research.	p.13
**OTHER INFORMATION**			
Registration and protocol	24a	Provide registration information for the review, including register name and registration number, or state that the review was not registered.	p.2
	24b	Indicate where the review protocol can be accessed, or state that a protocol was not prepared.	p.2
	24c	Describe and explain any amendments to information provided at registration or in the protocol.	p.2
Support	25	Describe sources of financial or non-financial support for the review, and the role of the funders or sponsors in the review.	p.14
Competing interests	26	Declare any competing interests of review authors.	p.14
Availability of data, code and other materials	27	Report which of the following are publicly available and where they can be found: template data collection forms; data extracted from included studies; data used for all analyses; analytic code; any other materials used in the review.	NA

Table S.2 – Search key, selection process and data collection process

Search Key	The same search key was used in all three databases (MEDLINE, Embase, CENTRAL):<br><br>(diabetes OR diab* OR prediabetes OR "pre-diabetes" OR "glucose tolerance" OR "glucose tolerant" OR "glucose intolerance" OR "glucose intolerant" OR "glucose control" OR "glucose metabolism" OR glucose homeostasis" OR hyperinsulinaemia OR hyperinsulinemia OR hyperglycaemia OR hyperglycemia OR dysglycaemia OR dysglycemia OR "insulin resistance" OR "insulin resistant" OR "endocrine dysfunction")<br>AND acute<br>AND pancreatitis
Selection process	Search results were exported to a citation manager software, EndNote X9.(1) Duplicates were first removed automatically by the software and then manually by a single investigator (OJZ).<br><br>Two independent reviewers (OJZ and AK) screened the resulting pool by predetermined criteria. Title and abstract selection were completed inclusively, and Cohen's kappa coefficient (κ) was calculated to assess the inter-reviewer agreement.(2) Discrepancies were settled by reaching a consensus after discussion.<br><br>The full-text selection was conducted in the same manner; in addition, any exclusions made in this phase were documented. Librarians, first and/or last authors and journal editors were contacted when the primary reviewers could not locate records. Studies of the same population were identified, in case they overlapped for exposure and outcome the article with the larger sample size was retained.
Data collection process	Two independent reviewers (DD and LH) extracted data into a standardized Excel sheet.(3) An independent reviewer (OJZ) assessed the validity of the data extracted and checked for missing data. In case of disagreements, a fourth independent reviewer (MFJ) made the final decision. When relevant data was not reported, authors were contacted for further information.

Table S.3 – List of prognostic factors

The list of prognostic factors mentioned in the included studies in relation to new-onset prediabetes, diabetes or prediabetes/diabetes. AP – acute pancreatitis, ERCP – Endoscopic retrograde cholangiopancreatography

**AP related factors**
**Laboratory parameters**<br>blood type, serum calcium, lactate dehydrogenase, random blood glucose, fasting blood glucose, stress hyperglycemia, creatine kinase levels, white blood cell count, neutrophils, total bilirubin, total cholesterol, triglyceride, amylase, fecal elastase, alanine aminotransferase, aspartate aminotransferase, gamma-glutamyl transferase
**Etiology**<br>Alcoholic, biliary, hypertriglyceridemic, idiopathic, post-ERCP
**Severity**<br>Severe, moderately severe, mild
**Recurrence**<br>≥2 AP episodes, ≥3 AP episodes
**Length of hospital stay**<br>≥7 days, ≥14 days
**Critical care admission**
**Complications**
**Organ failure**<br>≥1 organ, ≥2 organs<br>Acute kidney injury, acute respiratory distress syndrome
**Necrosis**
**Necrosis location**<br>Head, body, tail, whole
**Necrosis extent**<br><30%, 30-50%, >50%
**Infected necrosis**
**Pseudocyst**
**Pseudocyst size**<br>≤10 cm, >10 cm
**Other complications**<br>Sepsis, intra-abdominal infection
**Demographic factors**
**Age**
**Gender**
**Ethnicity**<br>European, Maori, Pacific Islander, Asian, Hispanic
**Socioeconomic deprivation**<br>1st, 2nd, 3rd, 4th quartile
**Income**<br>1st, 2nd, 3rd, 4th quartile<br><15,000; 15,000-22,798; ≥22,798 Taiwan New Dollars
**Lifestyle**
**Alcohol consumption**
**Smoking**

**Physical activity**
**Diet**
**Vitamins and medications**
**Comorbidities**
**Obesity**<br>Overweight, obese, abdominal obesity
**Hyperlipidemia**
**Liver disease**<br>Non-alcoholic fatty liver disease, alcoholic liver disease, fatty liver, liver cirrhosis, Hepatitis C
**Hypertension**
**Cardiovascular disease**
**Other comorbidities**<br>Charlson Comorbidity Index, chronic obstructive pulmonary disease, peptic ulcer disease, inflammatory bowel disease, gout, metabolic syndrome, depression

Table S.4 – Data synthesis and statistical analysis

Odds ratios (OR) for each risk factor in every study	For outcomes, odds ratio (OR) with 95% confidence interval (CI) was used for the effect size measure. To calculate the odds ratio, the total number of patients and those with the event of interest (in each group separately) were extracted from each study. If the raw numbers were not given, but were explicitly calculatable (e.g., OR, total numbers and one of the event count), the raw data were calculated.
Pooled OR	Mantel-Haenszel method.(4-6) As we anticipated considerable between-study heterogeneity, random-effects model meta-analysis was performed for each prognostic factor with at least three studies of the same outcome.
Visualization	Forest plots
Zero cell counts	Exact Mantel-Haenszel method without continuity correction(7, 8)
Correction of the pooled odds ratio's confidence interval	Hartung-Knapp adjustment was applied with a minimum of 5 studies(9, 10)
Tau (τ^2) estimation (heterogeneity variance)	Paule-Mandel method was applied to estimate the variance(11, 12) with the Q profile method for confidence interval(12)
I^2 calculation (between-study heterogeneity)	Higgins&Thompson's I^2 statistics with 95% confidence interval.(13) Interpretation of I^2 statistics according to the Cochrane handbook: <30% – low, 30% to 60% – moderate, 50% to 90% – substantial and 75% to 100% – considerable degree of heterogeneity.(14)
Leave-one-out analysis (outlier and influential analyses)	If more than 5 studies were included in the analysis, sensitivity analysis was performed following the recommendations of Harrer et al.(15) and Viechtbauer and Cheung.(16) Studies were marked influential when any of the following measures were influential: effect size, 95% confidence interval, I2, the studentized residuals, the difference in fits, Cook's distance, the covariance ratio, the value of the hat matrix.
Publication bias	Egger's test (at significance level 10% as small study number) using the Harbord method.(17) The analyses results should be (and were)

	critically handled while the study number was below 10, and the study effects showed high heterogeneity.
Sub-group analysis	Subgroup analysis was performed, separating studies according to their follow-up duration into: <1 year, 1-3 years, 3-6 years, >13 years categories to account for the varying follow-up length in the individual studies. These cut-off values were based on the available follow-up lengths in the eligible studies and a previous meta-analysis,(18) which found that the prevalence of new-onset PD/DM increased significantly within the 1st year post-AP and after 5 years, remaining reasonably stable between 1-3 and 3-5 years.<br><br>Fixed-effects "plural" model (aka. mixed-effects model). We assumed that all subgroup share a common Tau (τ2) value as we did not anticipate high difference in the between-study heterogeneity in the subgroups and the study number is relatively small in some subgroup. If applicable, to asses the difference between the subgroups a Cochrane Q test was used between subgroups(15). The null hypothesis was rejected on a 5% significance level.
Statistical software	R statistical software v4.2.1(19)<br>*meta* package v5.5.0(20)<br>*dmetar* package v0.0.9000(21)

RESULTS: Forest plots of risk factor & outcome pairs

Figure S.1 – Necrosis & risk of A) diabetes and B) prediabetes/ diabetes

A)

Study	Exposed DM	Exposed necrossis present	Control DM	Control necrosis absent	OR	95%-CI	Weight
Takeyama, 2009	7	98	58	401	0.45	[0.20; 1.03]	20.0%
Zhang et al., 2023	6	36	62	784	2.33	[0.93; 5.81]	19.3%
Bharmal et al., 2022a	1	6	7	114	3.06	[0.31; 29.86]	9.7%
Man et al., 2022	5	88	3	220	4.36	[1.02; 18.64]	14.9%
Robertson et al., 2023	34	209	4	133	6.27	[2.17; 18.10]	18.1%
Patra & Das, 2021	16	71	1	26	7.27	[0.91; 57.92]	10.8%
Tu et al., 2017	34	89	0	24	30.46	[1.79; 517.19]	7.3%
**Random effect**	**103**	**597**	**135**	**1702**	**3.09**	**[0.98; 9.72]**	**100.0%**

I^2 = 74% [45%; 88%], τ = 0.96

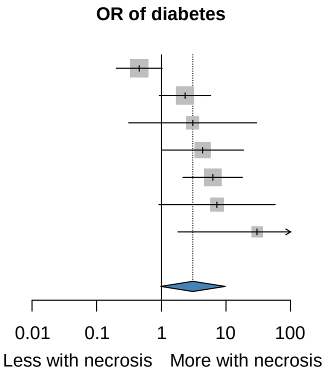

B)

Study	Exposed DM	Exposed necrosis present	Control DM	Control necrosis absent	OR	95%-CI	Weight
Tu et al., 2018	101	156	53	100	1.63	[0.98; 2.72]	25.6%
Li et al. 2021	4	5	32	67	4.38	[0.46; 41.22]	9.9%
Akbar et al., 2020	25	58	4	28	4.55	[1.40; 14.78]	18.6%
Garip et al., 2013	17	25	16	71	7.30	[2.67; 20.02]	20.5%
Yu et al., 2020	118	152	32	209	19.20	[11.23; 32.81]	25.4%
**Random effect**	**265**	**396**	**137**	**475**	**5.53**	**[1.59; 19.21]**	**100.0%**

I^2 = 91% [81%; 95%], τ = 0.85

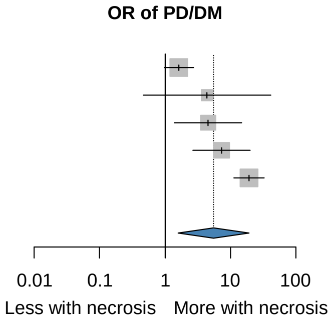

Figure S.1 Pancreatic necrosis as a complication of acute pancreatitis is associated with an increased odds of developing A) diabetes, B) prediabetes and diabetes. OR – odds ratio, CI – confidence interval, DM – diabetes mellitus, PD/DM – prediabetes and diabetes

Figure S.2 – Necrosis >30% of pancreas & risk of prediabetes/ diabetes

Study	Exposed PD/DM	Exposed >30%	Control PD/DM	Control <30%	OR	95%-CI	Weight
Tu et al., 2017	85	115	17	31	2.33	[1.03; 5.30]	34.2%
Akbar et al., 2022	21	49	8	37	2.72	[1.03; 7.14]	33.0%
Yu et al., 2020	103	110	15	42	26.49	[9.82; 71.44]	32.8%
**Random effect**	**209**	**274**	**40**	**110**	**5.44**	**[0.19; 157.71]**	**100.0%**

I^2 = 87% [64%; 96%], τ = 1.27

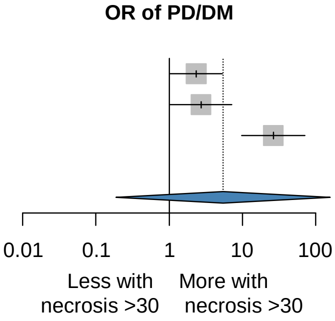

Figure S.2 Necrosis affecting over 30% of the pancreas is associated with an increased odds of developing prediabetes and diabetes. OR – odds ratio, CI – confidence interval, PD/DM – prediabetes and diabetes

Figure S.3 – Necrosis >50% of pancreas & risk of diabetes

Study	Exposed DM	Exposed >50%	Control DM	Control <50%	OR	95%-CI	Weight
Tu et al., 2017	51	89	17	57	3.16	[1.56; 6.40]	44.4%
Robertson et al., 2023	23	105	15	268	4.73	[2.36; 9.49]	45.6%
Man et al., 2022	3	26	5	282	7.23	[1.62; 32.17]	9.9%
**Random effect**	**77**	**220**	**37**	**607**	**4.12**	**[1.83; 9.30]**	**100.0%**

I^2 = 0% [0%; 90%], τ = 0

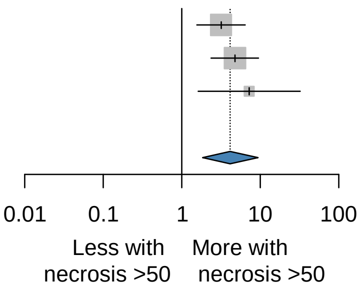

Figure S.3 Necrosis affecting over 50% of the pancreas is associated with an increased odds of developing diabetes. OR – odds ratio, CI – confidence interval, DM – diabetes mellitus

Figure S.4 – Organ failure & risk of A) diabetes and B) prediabetes/ diabetes

A)

Study	Exposed DM	Exposed organ failure	Control DM	Control no organ failure	OR	95%-CI	Weight
Robertson et al., 2023	10	77	28	260	1.24	[0.57; 2.67]	26.1%
Halonen et al., 2003	25	53	35	92	1.45	[0.73; 2.88]	26.9%
Patra & Das, 2021	7	16	9	74	5.62	[1.68; 18.82]	21.9%
Ma et al., 2019	11	56	11	560	12.20	[5.01; 29.69]	25.0%
**Random effect**	**53**	**202**	**83**	**986**	**3.19**	**[0.55; 18.64]**	**100.0%**

I^2 = 85% [62%; 94%], τ = 1.01

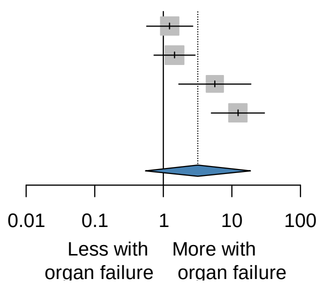

B)

Study	Exposed PD/DM	Exposed organ failure	Control PD/DM	Control no organ failure	OR	95%-CI	Weight
Akbar et al., 2022	9	24	20	62	1.26	[0.47; 3.37]	23.3%
Tu et al., 2018	62	92	92	164	1.62	[0.95; 2.76]	36.9%
Yu et al., 2020	84	137	66	224	3.79	[2.42; 5.94]	39.8%
**Random effect**	**155**	**253**	**178**	**450**	**2.14**	**[0.51; 9.06]**	**100.0%**

I^2 = 74% [14%; 92%], τ = 0.48

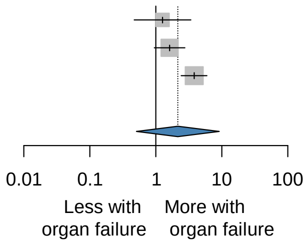

Figure S.4 Organ failure as a complication of acute pancreatitis is associated with an increased odds of developing A) diabetes, B) prediabetes and diabetes. OR – odds ratio, CI – confidence interval, DM – diabetes mellitus, PD/DM – prediabetes and diabetes

Figure S.5 – AP etiology: hypertriglyceridemic & risk of prediabetes/ diabetes

Study	Exposed PD/DM	Exposed HTG	Control PD/DM	Control non-HTG	OR	95%-CI	Weight
Yu et al., 2020	61	130	89	231	1.41	[0.91; 2.18]	41.6%
Yuan et al., 2017	14	32	55	278	3.15	[1.48; 6.73]	38.3%
Wu et al., 2011	6	7	12	52	20.00	[2.19; 182.88]	20.1%
**Random effect**	**81**	**169**	**156**	**561**	**3.27**	**[0.17; 63.91]**	**100.0%**

I^2 = 74% [15%; 92%], τ = 1.05

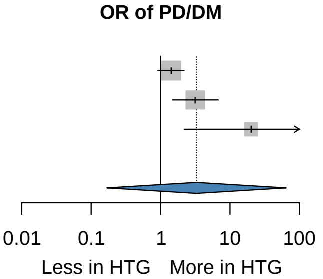

Figure S.5 The association between acute pancreatitis caused by hypertriglyceridemia and new-onset prediabetes and diabetes. AP – acute pancreatitis, HTG – hypertriglyceridemic, PD/DM – prediabetes and diabetes, OR – odds ratio, CI – confidence interval

Figure S.6 – AP etiology: alcoholic & risk of diabetes

Study	Exposed DM	Exposed alcoholic	Control DM	Control non-alcoholic	OR	95%-CI	Weight
**<1 year**							
Firkins et al., 2021	681	12977	1829	29841	0.85	[0.77; 0.93]	8.9%
Ma et al., 2019	18	86	105	530	1.07	[0.61; 1.88]	7.9%
**Random effect**	**699**	**13063**	**1934**	**30371**	**0.95**	**[0.22; 4.16]**	**16.8%**
I^2 = 0%, τ = 0.73							
**1-3 years**							
Vujasinovic et al., 2014	2	13	4	8	0.18	[0.02; 1.41]	3.3%
Robertson et al., 2023	10	128	28	209	0.55	[0.26; 1.17]	7.2%
Lv et al., 2022	3	68	108	1736	0.70	[0.22; 2.25]	5.7%
Angelini et al., 1984	2	14	2	13	0.92	[0.11; 7.67]	3.2%
Bojková et al., 2016	8	31	4	25	1.83	[0.48; 6.96]	5.2%
Bharmal et al., 2022a	3	26	5	94	2.32	[0.52; 10.44]	4.7%
Gold-Smith et al., 2020	8	21	4	72	10.46	[2.74; 39.91]	5.2%
**Random effect**	**36**	**301**	**155**	**2157**	**1.22**	**[0.39; 3.81]**	**34.5%**
I^2 = 69% [31%; 86%], τ = 0.73							
**3-6 years**							
Frey, 1969	45	141	31	138	1.62	[0.95; 2.76]	8.0%
Chowdhury et al., 2022	12	175	21	548	1.85	[0.89; 3.84]	7.3%
Ho et al., 2015	383	5728	235	6556	1.93	[1.63; 2.28]	8.8%
Andersson et al., 2010	4	10	5	29	3.20	[0.65; 15.70]	4.4%
Doepel et al., 1993	18	28	2	9	6.30	[1.09; 36.30]	4.0%
Patra & Das, 2021	8	21	5	74	8.49	[2.40; 30.08]	5.4%
Buscher et al., 1999	4	7	1	13	16.00	[1.27; 200.92]	2.5%
**Random effect**	**474**	**6110**	**300**	**7367**	**2.96**	**[1.45; 6.07]**	**40.5%**
I^2 = 42% [0%; 76%], τ = 0.73							
**>13 years**							
Takeyama, 2009	58	281	35	433	2.96	[1.89; 4.64]	8.2%
**Random effect**	**1267**	**19755**	**2424**	**40328**	**1.82**	**[1.09; 3.04]**	**100.0%**
**Prediction interval**						**[0.30; 10.98]**	

I^2 = 88% [83%; 92%], τ = 0.81

Residual heterogeneity: I^2 = 57% [22%; 76%], τ = 0.73

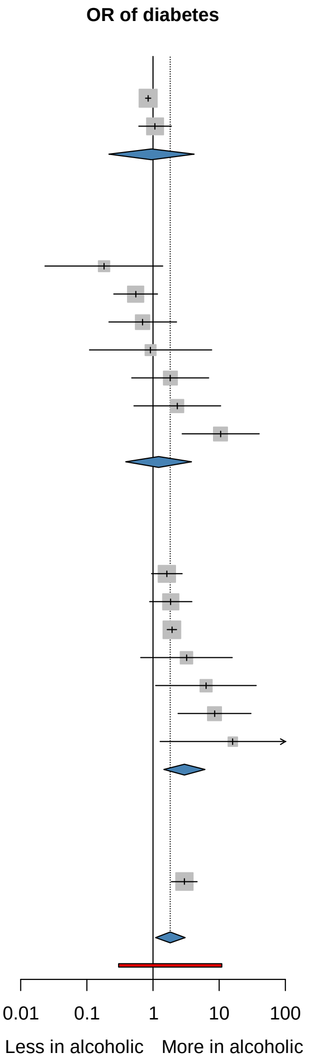

Figure S.6 Acute pancreatitis that was caused by excessive alcohol consumption is linked to a greater odds of developing diabetes and the effect increases with time to follow-up. AP – acute pancreatitis, OR – odds ratio, CI – confidence interval, DM – diabetes mellitus

Figure S.7 – AP etiology: alcoholic & risk of prediabetes/diabetes

Study	Exposed PD/DM	Exposed alcoholic	Control PD/DM	Control non-alcoholic	OR	95%-CI	Weight
Angelini et al., 1984	8	14	8	13	0.83	[0.18; 3.88]	7.7%
Pendharkar et al., 2018b	6	18	24	65	0.85	[0.28; 2.57]	13.5%
Wu et al., 2011	2	7	16	52	0.90	[0.16; 5.14]	6.2%
Yu et al., 2020	21	52	129	309	0.95	[0.52; 1.72]	30.7%
Yuan et al., 2017	4	15	65	295	1.29	[0.40; 4.18]	12.1%
Li et al. 2021	10	17	26	55	1.59	[0.53; 4.79]	13.5%
Buscher et al., 1999	5	7	6	13	2.92	[0.41; 20.90]	4.9%
Malecka-Panas et al., 2002	13	36	4	46	5.93	[1.73; 20.32]	11.3%
**Random effect**	**69**	**166**	**278**	**848**	**1.33**	**[0.77; 2.31]**	**100.0%**

I^2 = 20% [0%; 62%], τ = 0.29

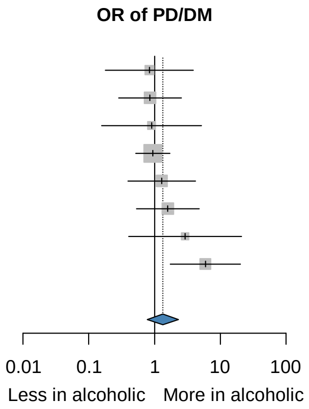

Figure S.7 The association between alcoholic acute pancreatitis etiology and new-onset prediabetes and diabetes**.** AP – acute pancreatitis, OR – odds ratio, CI – confidence interval, PD/DM – prediabetes and diabetes

Figure S.8 – AP etiology: biliary & risk of A) diabetes and B) prediabetes/ diabetes

A)

Study	Exposed DM	Exposed biliary	Control DM	Control non-biliary	OR	95%-CI	Weight
Hochman et al., 2006	0	11	8	14	0.03	[0.00; 0.67]	0.8%
Buscher et al., 1999	1	10	4	10	0.17	[0.01; 1.88]	1.2%
Boreham & Ammori, 2003	1	13	3	10	0.19	[0.02; 2.25]	1.2%
Man et al., 2022	3	204	5	104	0.30	[0.07; 1.26]	2.8%
Andersson et al., 2010	3	19	6	20	0.44	[0.09; 2.08]	2.5%
Chowdhury et al., 2022	12	386	21	337	0.48	[0.23; 1.00]	6.2%
Ho et al., 2015	235	6556	383	5728	0.52	[0.44; 0.61]	10.3%
Bojková et al., 2016	3	20	9	36	0.53	[0.13; 2.24]	2.8%
Walker et al., 2022b	79	820	153	928	0.54	[0.40; 0.72]	9.6%
Lv et al., 2022	44	971	67	833	0.54	[0.37; 0.80]	8.8%
Takeyama, 2009	11	121	82	593	0.62	[0.32; 1.21]	6.7%
Ma et al., 2019	48	263	75	353	0.83	[0.55; 1.24]	8.7%
Firkins et al., 2021	379	7181	2131	35637	0.88	[0.78; 0.98]	10.5%
Frey, 1969	17	65	59	214	0.93	[0.50; 1.75]	7.0%
Cho et al., 2020	171	2775	387	6696	1.07	[0.89; 1.29]	10.2%
Bharmal et al., 2022a	5	64	3	56	1.50	[0.34; 6.57]	2.7%
Robertson et al., 2023	19	129	19	208	1.72	[0.87; 3.38]	6.6%
Vujasinovic et al., 2014	3	5	3	16	6.50	[0.73; 57.83]	1.4%
**Random effect**	**1034**	**19613**	**3418**	**51793**	**0.70**	**[0.52; 0.95]**	**100.0%**
**Prediction interval**						**[0.26; 1.87]**	

I^2 = 75% [61%; 84%], τ = 0.44

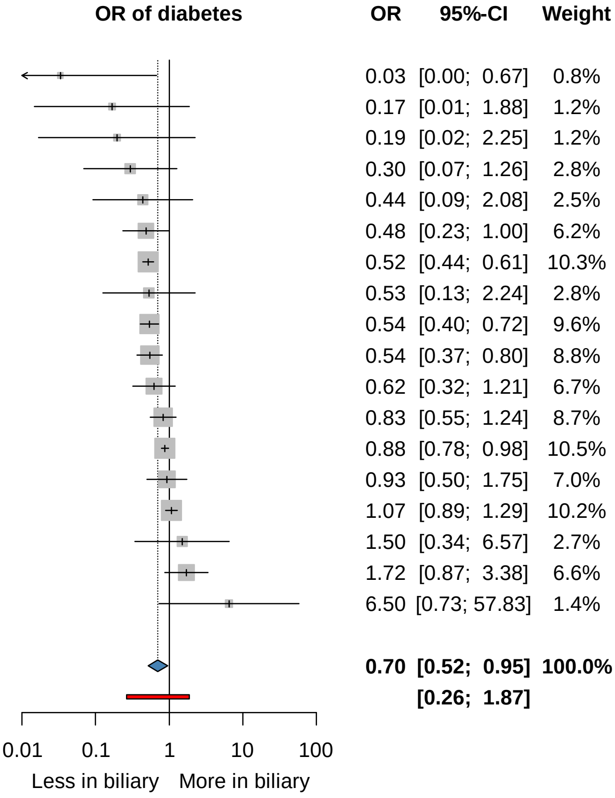

B)

Study	Exposed PD/DM	Exposed biliary	Control PD/DM	Control non-biliary	OR	95%-CI	Weight
Wu et al., 2011	10	42	8	17	0.35	[0.11; 1.15]	5.4%
Malecka-Panas et al., 2002	4	29	13	53	0.49	[0.14; 1.68]	5.1%
Yuan et al., 2017	28	153	41	157	0.63	[0.37; 1.09]	26.1%
Buscher et al., 1999	5	10	6	10	0.67	[0.11; 3.92]	2.5%
Yu et al., 2020	63	166	87	195	0.76	[0.50; 1.16]	43.2%
Pendharkar et al., 2018b	13	37	17	46	0.92	[0.37; 2.28]	9.4%
Li et al. 2021	14	26	22	46	1.27	[0.49; 3.34]	8.3%
**Random effect**	**137**	**463**	**194**	**524**	**0.72**	**[0.55; 0.95]**	**100.0%**

I^2 = 0% [0%; 71%], τ = 0

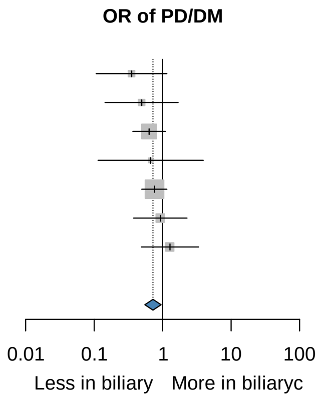

Figure S.8 The association between biliary acute pancreatitis and new-onset A) diabetes, B) prediabetes and diabetes. AP – acute pancreatitis, OR – odds ratio, CI – confidence interval, DM – diabetes mellitus, PD/DM – prediabetes and diabetes

Figure S.9 – AP etiology: idiopathic & risk of diabetes

Study	Exposed DM	Exposed idiopathic	Control DM	Control known etiology	OR	95%-CI	Weight
Ma et al., 2019	24	221	81	395	0.47	[0.29; 0.77]	27.5%
Takeyama, 2009	16	162	77	552	0.68	[0.38; 1.19]	23.8%
Frey, 1969	8	30	68	249	0.97	[0.41; 2.28]	14.3%
Lv et al., 2022	37	525	74	1279	1.23	[0.82; 1.86]	31.9%
Bojková et al., 2016	1	4	11	52	1.24	[0.12; 13.15]	2.5%
**Random effect**	**86**	**942**	**311**	**2527**	**0.79**	**[0.46; 1.37]**	**100.0%**

I^2 = 58% [0%; 84%], τ = 0.28

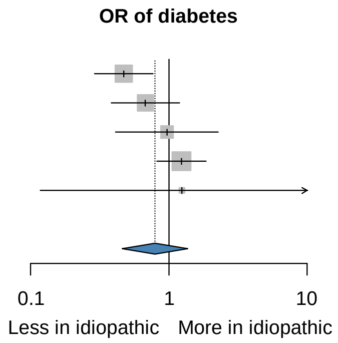

Figure S.9 The association between idiopathic acute pancreatitis and new-onset diabetes. AP – acute pancreatitis, OR – odds ratio, CI – confidence interval, DM – diabetes mellitus

Figure S.10 – Number of AP episodes ≥3 & risk of diabetes

Study	Exposed DM	Exposed >3 episodes	Control DM	Control 1-2 episodes	OR	95%-CI	Weight
Ho et al., 2015	149	1622	469	10662	2.20	[1.81; 2.66]	80.6%
Man et al., 2022	1	18	7	290	2.38	[0.28; 20.45]	4.1%
Tu et al., 2023	12	24	10	64	5.40	[1.90; 15.38]	15.3%
**Random effect**	**162**	**1664**	**486**	**11016**	**2.53**	**[0.95; 6.74]**	**100.0%**

I^2 = 27% [0%; 92%], τ = 0.23

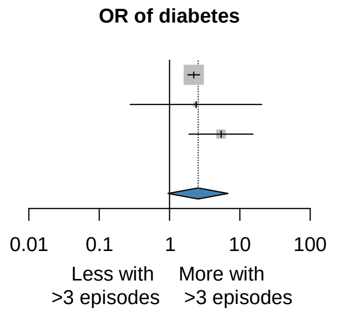

Figure S.10 The association between the number of acute pancreatitis episodes and new-onset diabetes. OR – odds ratio, CI – confidence interval, DM – diabetes mellitus

Figure S.11 – Comorbidity: obesity & risk of diabetes

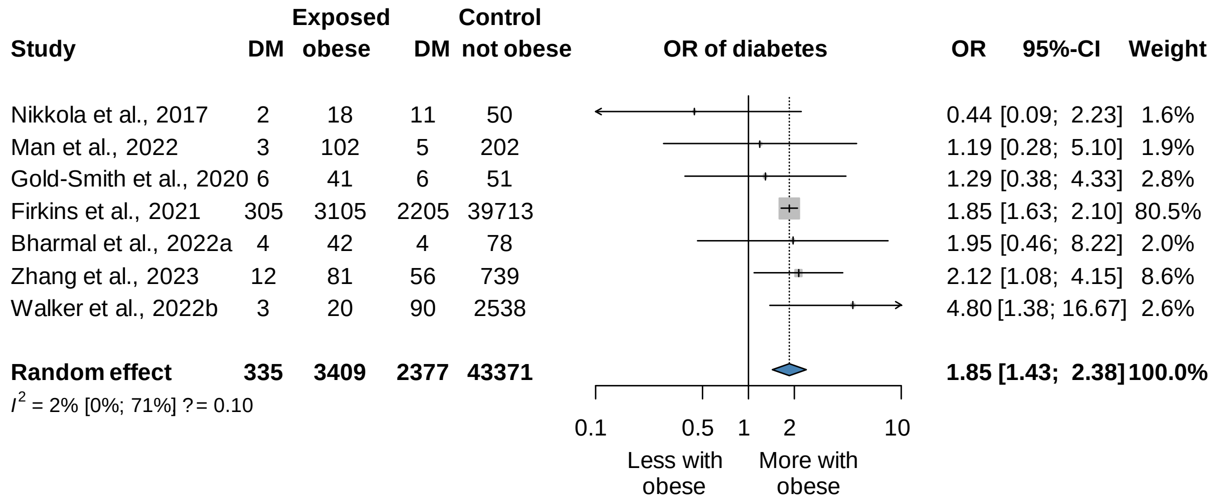

Figure S.21 The association between obesity and new-onset diabetes after acute pancreatitis. OR – odds ratio, CI – confidence interval, DM – diabetes mellitus

Figure S.12 – Comorbidity: overweight and obese & risk of diabetes

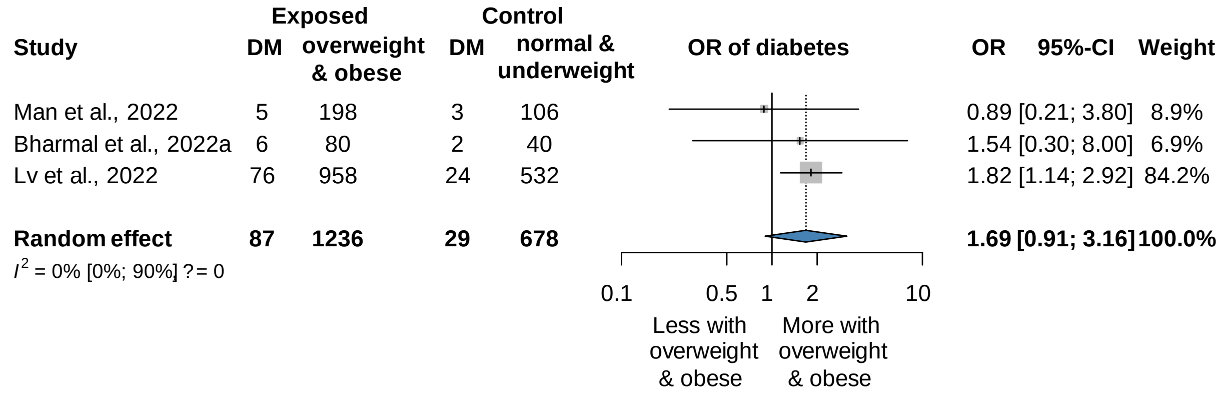

Figure S.32 The association between being overweight or obese and new-onset diabetes after acute pancreatitis. OR – odds ratio, CI – confidence interval, DM – diabetes mellitus

Figure S.13 – Comorbidity: dyslipidemia & risk of diabetes

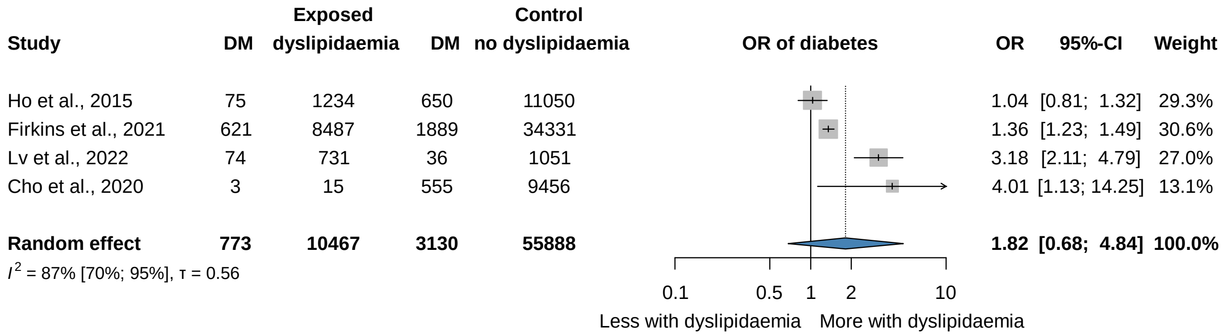

Figure S.13 The association between dyslipidemia and new-onset diabetes after acute pancreatitis. OR – odds ratio, CI – confidence interval, DM – diabetes mellitus

Figure S.14 – Comorbidity: liver cirrhosis & risk of diabetes

Study	Exposed DM	Exposed cirrhosis	Control DM	Control no cirrhosis	OR	95%-CI	Weight
Ho et al., 2015	31	522	694	11762	1.01	[0.69; 1.46]	45.6%
Chowdhury et al., 2022	6	41	27	682	4.16	[1.61; 10.73]	36.7%
Hietanen et al., 2014	4	22	1	40	8.67	[0.90; 83.17]	17.6%
**Random effect**	**41**	**585**	**722**	**12484**	**2.48**	**[0.18; 34.25]**	**100.0%**

I^2 = 81% [39%; 94%], τ = 0.88

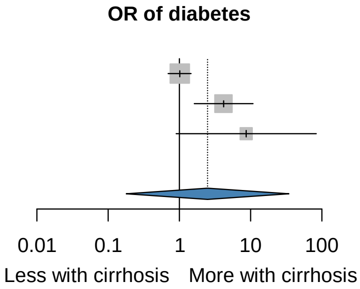

Figure S.14 The association between liver cirrhosis and new-onset diabetes after acute pancreatitis. OR – odds ratio, CI – confidence interval, DM – diabetes mellitus

Figure S.15 – Comorbidity: other liver diseases & risk of diabetes

Study	Exposed DM	Exposed liver disease	Control DM	Control no liver disease	OR	95%-CI	Weight
Firkins et al., 2021	69	1311	2441	41507	0.89	[0.70; 1.14]	35.3%
Cho et al., 2021	17	154	694	10716	1.79	[1.08; 2.98]	31.6%
Lv et al., 2022	79	788	32	1016	3.43	[2.25; 5.22]	33.0%
**Random effect**	**165**	**2253**	**3167**	**53239**	**1.73**	**[0.32; 9.46]**	**100.0%**

I^2 = 94% [84%; 97%], τ = 0.65

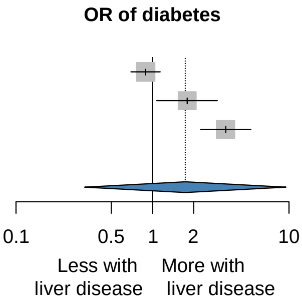

Figure S.15 The association between liver diseases (other than liver cirrhosis) and new-onset diabetes after acute pancreatitis. OR – odds ratio, CI – confidence interval, DM – diabetes mellitus

Figure S.16 – Comorbidity: chronic kidney disease & risk of diabetes

Study	Exposed DM	Exposed CKD	Control DM	Control no CKD	OR	95%-CI	Weight
Robertson et al., 2023	2	15	36	322	1.22	[0.27; 5.64]	0.4%
Chowdhury et al., 2022	1	18	32	705	1.24	[0.16; 9.59]	0.2%
Firkins et al., 2021	625	6104	1885	36714	2.11	[1.92; 2.32]	99.4%
**Random effect**	**628**	**6137**	**1953**	**37741**	**2.10**	**[1.85; 2.38]**	**100.0%**

I^2 = 0% [0%; 90%], τ = 0

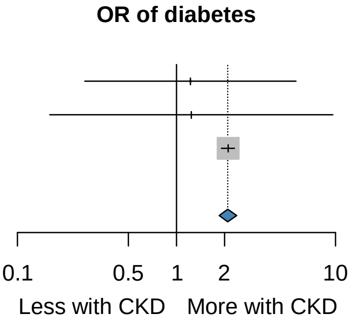

Figure S.16 The association between chronic kidney disease and new-onset diabetes after acute pancreatitis. OR – odds ratio, CI – confidence interval, DM – diabetes mellitus, CKD – chronic kidney disease

Figure S.17 – Comorbidity: hypertension & risk of diabetes

Study	DM	Exposed hypertension	DM	Control no hypertension	OR	95%-CI	Weight
Robertson et al., 2023	10	93	28	244	0.93	[0.43; 2.00]	8.6%
Lv et al., 2022	31	452	80	1352	1.17	[0.76; 1.80]	18.7%
Ho et al., 2015	196	3581	529	11755	1.23	[1.04; 1.45]	34.2%
Firkins et al., 2021	1615	21805	895	21013	1.80	[1.65; 1.96]	38.5%
**Random effect**	**1852**	**25931**	**1532**	**34364**	**1.38**	**[0.91; 2.08]**	**100.0%**

I^2 = 85% [64%; 94%], τ = 0.20

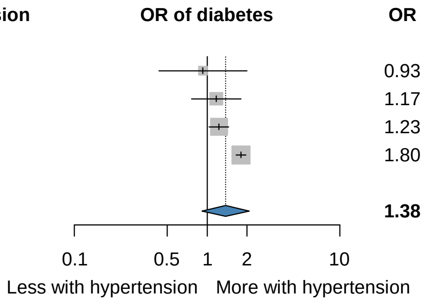

Figure S.17 The association between hypertension and new-onset diabetes after acute pancreatitis. OR – odds ratio, CI – confidence interval, DM – diabetes mellitus

Figure S.18 – Comorbidity: cardiovascular disease & risk of diabetes

Study	DM	Exposed CVD	DM	Control no CVD	OR	95%-CI	Weight
Lv et al., 2022	5	165	106	1639	0.45	[0.18; 1.12]	13.6%
Robertson et al., 2023	2	26	36	311	0.64	[0.14; 2.81]	6.5%
Ho et al., 2015	75	1635	650	10649	0.74	[0.58; 0.94]	35.9%
Chowdhury et al., 2022	1	27	32	696	0.80	[0.10; 6.07]	3.7%
Firkins et al., 2021	684	8993	1826	33825	1.44	[1.32; 1.58]	40.3%
**Random effect**	**767**	**10846**	**2650**	**47120**	**0.90**	**[0.50; 1.61]**	**100.0%**

I^2 = 87% [73%; 94%], τ = 0.33

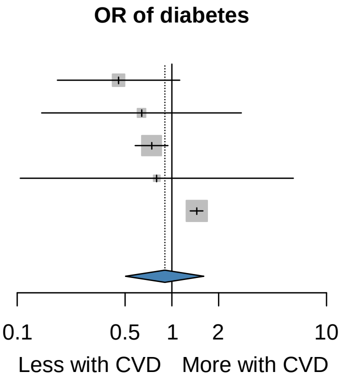

Figure S.18 The association between cardiovascular disease and new-onset diabetes after acute pancreatitis. OR – odds ratio, CI – confidence interval, DM – diabetes mellitus, CVD – cardiovascular disease

Figure S.19 – Alcohol consumption & risk of A) diabetes B) prediabetes/diabetes

A)

Study	DM	Exposed drinking	DM	Control no drinking	OR	95%-CI	Weight
Robertson et al., 2023	20	206	18	131	0.68	[0.34; 1.33]	18.9%
Lv et al., 2022	28	463	83	1341	0.98	[0.63; 1.52]	29.5%
Nikkola et al., 2017	6	26	7	42	1.50	[0.44; 5.09]	7.9%
Cho et al., 2021	121	1299	590	9571	1.56	[1.27; 1.92]	43.8%
**Random effect**	**175**	**1994**	**698**	**11085**	**1.16**	**[0.63; 2.12]**	**100.0%**

I^2 = 63% [0%; 87%], τ = 0.27

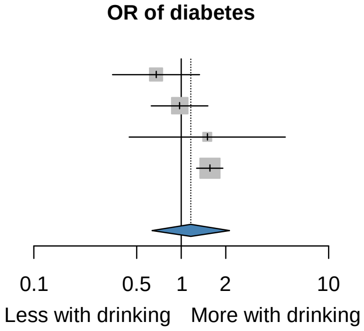

B)

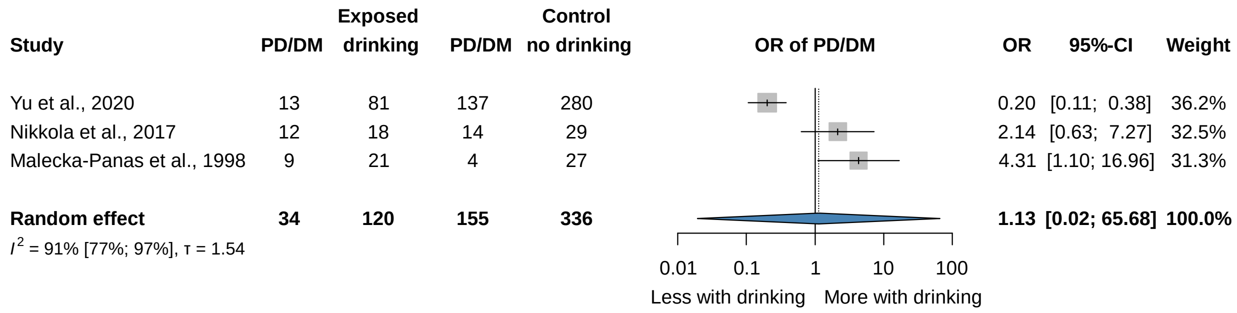

Figure S.19 The association between alcohol consumption and new-onset A) diabetes, B) prediabetes and diabetes. AP – acute pancreatitis, OR – odds ratio, CI – confidence interval, DM – diabetes mellitus, PD/DM – prediabetes and diabetes

Figure S.20 – Smoking & risk of A) diabetes B) prediabetes/diabetes

A)

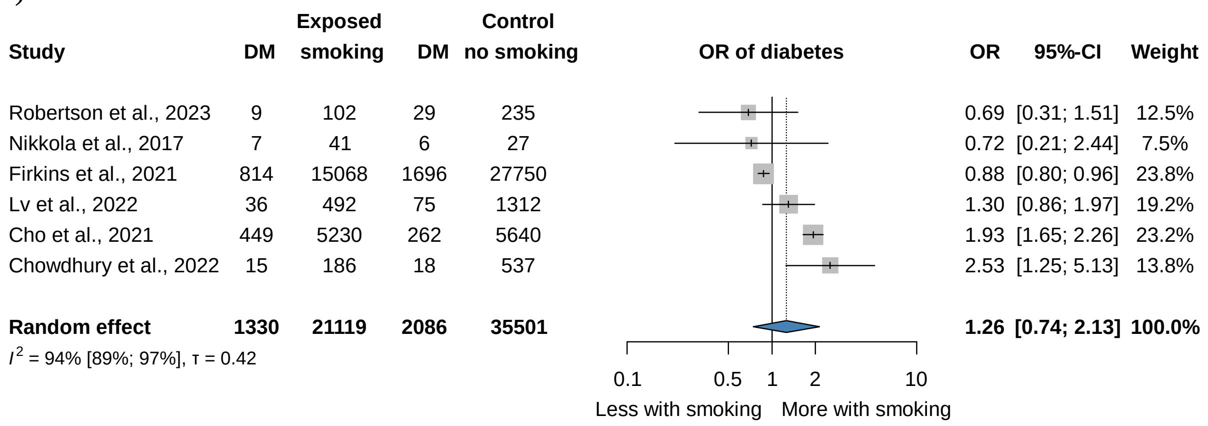

B)

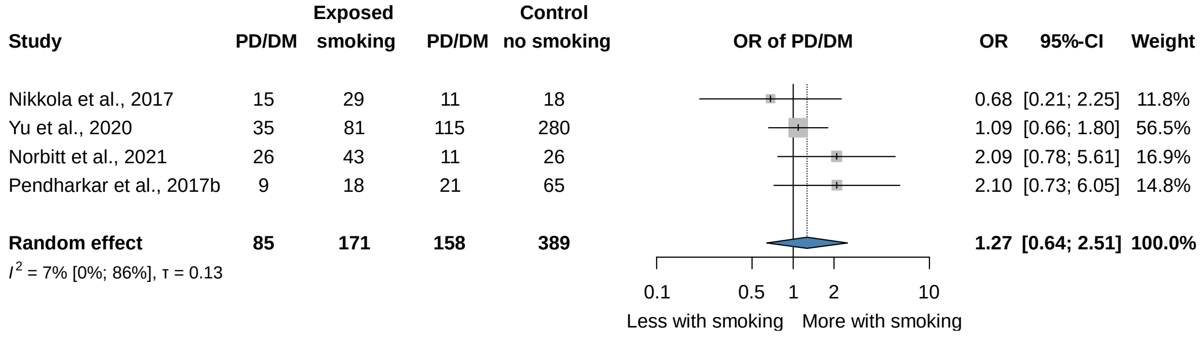

Figure S.20 The association between smoking and new-onset A) diabetes, B) prediabetes and diabetes. OR – odds ratio, CI – confidence interval, DM – diabetes mellitus, PD/DM – prediabetes and diabetes

Figure S.21 – Sex & risk of A) diabetes B) prediabetes/diabetes

A)

Study	Exposed DM	Exposed male	Control DM	Control female	OR	95%-CI	Weight
Uomo et al., 2010	0	17	6	23	0.08	[0.00; 1.47]	1.5%
Robertson et al., 2023	17	203	21	134	0.49	[0.25; 0.97]	10.2%
Boreham & Ammori, 2003	2	13	2	10	0.73	[0.08; 6.31]	2.5%
Burge & Gabaldon-Bates, 2003	59	495	39	392	1.22	[0.80; 1.88]	12.7%
Cho et al., 2020	306	4506	252	4965	1.36	[1.15; 1.62]	14.8%
Lv et al., 2022	78	1139	33	665	1.41	[0.93; 2.14]	12.8%
Man et al., 2022	5	167	3	141	1.42	[0.33; 6.05]	4.6%
Ho et al., 2015	503	8678	115	3606	1.87	[1.52; 2.30]	14.6%
Walker et al., 2022b	141	863	81	885	1.94	[1.45; 2.59]	14.0%
Chowdhury et al., 2022	23	363	10	360	2.37	[1.11; 5.05]	9.4%
Vujasinovic et al., 2014	6	17	0	4	5.09	[0.23; 110.24]	1.4%
Bharmal et al., 2022a	8	70	0	50	13.74	[0.77; 243.76]	1.5%
**Random effect**	**1148**	**16531**	**562**	**11235**	**1.41**	**[0.92; 2.14]**	**100.0%**
**Prediction interval**						**[0.44; 4.53]**	

I^2 = 61% [27%; 79%], τ = 0.49

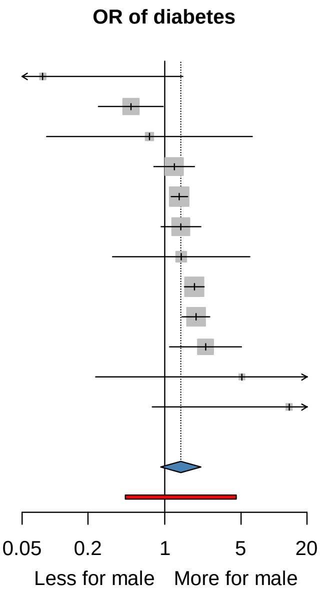

B)

Study	Exposed DM	Exposed male	Control DM	Control female	OR	95%-CI	Weight
Yuan et al., 2017	40	186	29	124	0.90	[0.52; 1.55]	20.1%
Yu et al., 2020	84	201	66	160	1.02	[0.67; 1.56]	30.1%
Pendharkar et al., 2018b	20	50	10	33	1.53	[0.60; 3.90]	7.6%
Li et al. 2021	26	48	10	24	1.65	[0.61; 4.45]	6.8%
Guo et al., 2022	111	317	42	175	1.71	[1.12; 2.59]	30.6%
Akbar et al., 2022	25	66	4	20	2.44	[0.73; 8.12]	4.7%
**Random effect**	**306**	**868**	**161**	**536**	**1.29**	**[0.91; 1.83]**	**100.0%**

I^2 = 18% [0%; 62%], τ = 0.12

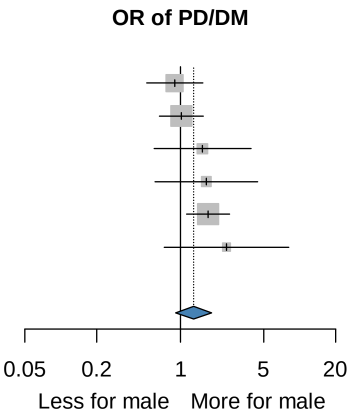

Figure S.214 The association between male sex and new-onset A) diabetes, B) prediabetes and diabetes following acute pancreatitis. OR – odds ratio, CI – confidence interval, DM – diabetes mellitus, PD/DM – prediabetes and diabetes

Figure S.22 – Age & risk of diabetes

Study	Exposed DM	Exposed >65 years	Control DM	Control <65 years	OR	95%-CI	Weight
Ho et al., 2015	107	2686	511	9598	0.74	[0.60; 0.91]	41.7%
Firkins et al., 2021	663	11158	1847	31660	1.02	[0.93; 1.12]	54.2%
Chowdhury et al., 2022	3	47	30	676	1.47	[0.43; 5.00]	4.1%
**Random effect**	**773**	**13891**	**2388**	**41934**	**0.90**	**[0.51; 1.60]**	**100.0%**

I^2 = 75% [16%; 92%], τ = 0.17

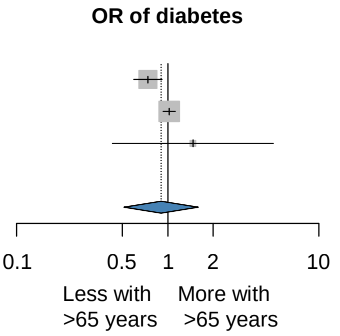

Figure S.225 The association between age and new-onset diabetes following acute pancreatitis. OR – odds ratio, CI – confidence interval, DM – diabetes mellitus

RESULTS: Qualitative analysis

Table S.5 – Qualitative summary

Qualitative summary of the association between the presence of prognostic factor and risk of impaired glucose homeostasis by comparison with the absence of prognostic factor in acute pancreatitis. PD – prediabetes, DM – diabetes mellitus, PD/DM – prediabetes and diabetes, OR – odds ratio, CI – confidence interval, AP – acute pancreatitis, ERCP – Endoscopic retrograde cholangiopancreatography, HTG – hypertriglyceridemic,

Prognostic factor	Outcome	Findings
**AP related factors**		
**Laboratory parameters**	DM, PD/DM	Yuan et al.(22) reported a positive association between AB blood type and new-onset DM, however Lv et al.(23) found no association between blood type and post-AP DM.<br><br>Patients who later developed DM or PD/DM had higher random blood glucose (22-24) and fasting plasma glucose levels (23, 25) during hospitalization than patients who remained normoglycemic. Stress hyperglycemia during hospitalization is associated with an increased risk of post-AP DM (OR: 4.41)(26). Baseline triglyceride (23, 25, 27, 28) and total cholesterol levels (23, 24, 27) were higher in patients who subsequently developed DM or PD/DM.<br><br>Yuan et al.(22) found that on admission decreased serum calcium was associated with new-onset PD/DM, however three other studies found no effect on PD/DM or DM development(23, 25, 27). Two studies reported a positive association with high lactate dehydrogenase levels and new-onset DM (23) and PD/DM(22), while two additional studies found no effect(25, 27). Yuan et al.(22) observed an increased risk of post-AP PD/DM in patients with elevated creatinine kinase levels, however the association was not supported by Zhang et al.(27).<br><br>Guo et al.(25) noted a positive association between increased white blood cell count and subsequently developed PD/DM, which was confirmed neither by Yuan et al.(22) nor Ma et al.(24). No association was found between neutrophil (22) or aspartate aminotransferase levels (22, 23, 27) and new-onset PD/DM or DM. One study reported an association between decreased alanine aminotransferase levels and subsequent post-AP DM(27), while additional two studies found no association(22, 23).<br><br>Elevated total bilirubin and gamma-glutamyl transferase levels were linked to an increased risk of developing PD/DM by one study(25). Guo et al.(25) found that patients with higher amylase levels were more likely to develop PD/DM. Two additional studies on the other hand reported lower amylase levels in patients developing new-onset

		DM(23, 27). One study found that fecal elastase-1 levels under 200 µg/g was associated with a greater risk (OR: 10.5) of developing PD/DM(29).
**Etiology**	PD, DM	No association was found between new-onset prediabetes and alcoholic etiology(30-33) or biliary etiology(31-33). Several studies excluded patients with post-ERCP AP(24, 31, 33-38). One study that examined the association between post-ERCP AP and DM development found no association(39). Two studies found an increased risk of developing DM after AP that was caused by HTG(24) or hyperlipidemia (defined as HTG or hypercholesterolemia)(23). Idiopathic AP was not associated with new-onset PD/DM(40, 41).
**Severity**	PD	Two studies found that severe AP and moderately severe AP were associated with an increased risk of developing prediabetes(42, 43).
**Length of hospital stay**	DM, PD/DM	Regarding length of hospital stay, three reports found no association with diabetes(23) or PD/DM(22), while one study showed increased odds (OR: 1.31; p=0.001) of diabetes when hospitalization exceeded 7 days(44).
**Critical care admission**	DM	One study found that 37% of the patients who later developed DM were admitted to intensive care unit compared to 26% of AP patients who remained normoglycemic(39). However, the association was not statistically significant (p=0.179). Another study noted a link between critical care admission and new-onset DM, which remained significant even after adjusting for multiple factors (HR: 2.37; p<0.001)(45).
**Complications**		
**Organ failure**	DM, PD/DM	Organ failure affecting two or more organs was not associated with post-AP DM compared to patients with single or no organ failure(39, 46). Some studies specified the type of organ failure. Neither acute kidney injury(47) nor need for dialysis(39) was associated with new-onset PD/DM and DM. The same studies found no association between acute respiratory distress syndrome(47) or mechanical ventilation(39) and PD/DM or DM development. Nonetheless, one study noted an increased risk (HR: 3.47; p<0.001) of post-AP DM with non-invasive ventilation(45).
**Necrosis**	PD	Tu, J. et al.(42) found that 69% of the patients who developed prediabetes had necrosis.
**Necrosis location**	PD, DM, PD/DM	Regarding the location of necrosis one study reported increased odds of developing DM when necrosis was in the tail region or dispersed in the whole pancreas (OR: 2.49; 1.52 respectively).(42) Yu et al.(48) found no statistically significant difference amongst the regions for PD and PD/DM incidence. However, necrosis was located in the tail region in the majority of patients developing PD/DM (48%) compared to 17%, 19% and 15% in the head, body and whole pancreas respectively. Huang et al.(28) observed that patients who had walled-off pancreatic necrosis in the tail region were more likely to develop post-AP DM (HR: 2.9; p=0.191), however the association was not statistically significant. Another study found no effect of pancreatic necrosis location on post-AP DM development(39).

**Necrosis extent**	PD, DM, PD/DM	One study explored the size of the necrotic area and prediabetes development(42). No association was found. On the other hand, a positive association was noted with >30% necrosis and DM development in two studies(42, 49). Necrosis affecting more than half of the pancreas was also significantly associated with an increased risk of PD/DM(42, 48).
**Infected necrosis**	PD, DM	Infected necrosis was found to be associated with an increased risk of developing diabetes by two studies: OR: 2.22(50), and OR: 4.61(42). No significant association was found with prediabetes.(42)
**Pseudocyst**	DM, PD/DM	Two small studies (n<60) found no association with pseudocyst formation and PD/DM (OR: 0.22; p=0.31)(40) or diabetes (OR: 0.75; p=0.73)(51). Three additional studies found an increased risk of DM development in patients with pseudocysts(44, 52, 53), which was statistically significant in the two larger studies(44, 52).
**Pseudocyst size**	PD/DM	Malecka-Panas et al.(54) noted that pseudocysts exceeding 10 cm increased the odds of developing PD/DM (OR: 1.81) compared to smaller cysts, however the association was not statistically significant (p>0.05).
Other c**omplication**s	DM	Analysis of a large American database revealed no significant association between diabetes and sepsis (OR: 1.34; p=0.111) or intra-abdominal infection (OR: 1.47; p=0.316).(44)
**Demographic factors**		
**Age**	DM, PD/DM	Two studies reported no association with age and PD/DM(22, 48), while two studies noted an association with diabetes, however of opposing effect. One study found the prevalence of new-onset diabetes was greater in participants below age 50 (5.6% vs 4.3%)(55), whereas the other study reported a prevalence of 6.6% in adults over 50 compared to younger adults (5.0%)(44).
**Gender**	PD	One study reported no effect of gender on the development of prediabetes following AP(33).
E**thnicity**	DM	One study found that Maori or Pacific Islander ethnicity was associated with increased odds (OR: 1.77) of developing diabetes compared to European or Asian origins(34). Two studies reported on Hispanic ethnicity with controversial results(56, 57).
**Socioeconomic deprivation**	DM	In a New Zealandic cohort, participants with the worst socioeconomic deprivation index had a greater incidence of diabetes (8.9%) compared to the least deprived patients (5.0%)(34).
I**ncome**	DM	In an American study the prevalence of new-onset diabetes in the lowest compared to the highest income quartiles was 7.2% and 4.9% respectively(44). A Taiwanese report found no association between income and diabetes following AP(55).
**Lifestyle**		
**Physical activity**	DM, PD/DM	Physical activity was not associated with diabetes(23, 35) or PD/DM(38).
**Diet**	DM	One study investigating the effect of dietary fat content on post-AP DM found no association(23). The study did not elaborate on the assessment of dietary intake, nor specified the cut offs used to define the low fat and high fat groups.

**Vitamins and medications**	DM, PD/DM	One study reported an association between beta cell function and consumption of certain vitamins (alpha-carotene, beta-carotene, total carotene and vitamin B3) in patients with post-AP PD/DM(58). Thiruvengadam et al.(59) found that regular statin users were significantly less likely to develop post-AP DM compared to nonusers (HR: 0.58; p<0.001).
**Comorbidities**		
**Obesity**	PD, DM, PD/DM	One study(31) reported that in their cohort 50% of the obese patients developed prediabetes compared to 13% of normal weight patients. Of the four studies examining the relationship between new-onset PD/DM and obesity(22, 29, 40, 48), only one study found a significant association(40). It is noteworthy that this study included the highest proportion of AP patients with biliary etiology (71%(40) vs 50%(22), 45%(48) and 0%(29)).<br><br>Two studies compared the risk of developing PD/DM in overweight and obese patients to normal and underweight patients with inconclusive results(22, 25). One of the studies found no effect(22), whereas the other reported a statistically significant positive association (HR: 2.05 p<0.001)(25).<br><br>Trikudanathan et al.(60) reported that abdominal obesity was associated with a significantly increased risk of developing DM even after adjusting for age, sex and multi-organ failure (OR: 3.01; p=0.02).
**Hyperlipidemia**	PD/DM	Hyperlipidemia at index admission was associated with a significantly increased odds of subsequently developed PD/DM in two Chinese studies (OR: 30.7; 95% CI: 1.53-615.21 (40) and HR: 2.52; 95% CI: 1.53-4.14)(22).
**Liver disease**	PD/DM	Fatty liver was associated with a significantly greater risk of developing PD/DM (HR: 1.87; 95% CI: 1.16-3.01)(22).
**Hypertension**	PD/DM	One study found that the incidence of PD/DM was 29.9% in patients with hypertension compared to 18.2% in non-hypertensive patients(22). Guo et al.(25) found no such association.
**Cardiovascular disease**	PD/DM	Two studies observed no link between cardiovascular disease and new-onset PD/DM after AP(22, 25).
**Other comorbidities**	DM	There was no significant association between new-onset diabetes and the Charlson comorbidity index(45, 55), peptic ulcer disease (55) or depression (OR: 0.82; p=0.054)(44). A small study (n=35) noted higher proportion of post-AP DM in patients without inflammatory bowel disease(61). Gout was associated a significantly increased risk of developing DM even after adjusting for several factors (HR: 1.58; 95% CI: 1.04-2.41)(52). One study reported an increased risk post-AP DM with pre-existing metabolic syndrome (HR: 1.54; p=0.041)(45). Firkins et al.(44) reported a significant association between new-onset diabetes and chronic obstructive pulmonary disease (OR: 1.25; p=0.016). The association was absent in a smaller study(39).

RESULTS: Risk of bias assessment

Figure S.23 – Risk of bias assessment: prediabetes

A) overall assessment

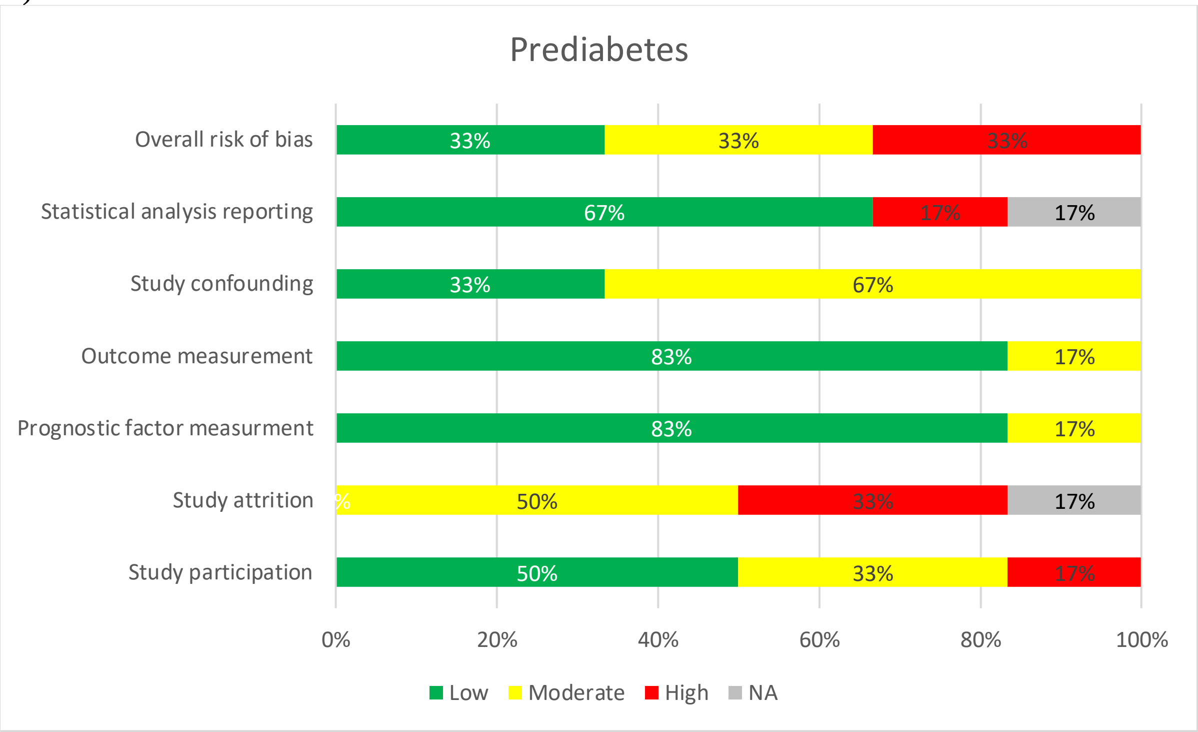

B) scoring of individual studies

Study	Study participation	Study attrition	Prognostic factor measurement	Outcome measurement	Study confounding	Statistical analysis reporting	Overall risk of bias
Angelini et al., 1984	H	H	L	M	M	NA	H
Bharmal et al., 2020	M	NA	L	L	M	L	M
Bharmal et al., 2022b	L	M	L	L	L	L	L
Buscher et al., 1999	L	M	L	L	M	L	M
Miko et al., 2022b	M	H	M	L	M	H	H
Tu et al., 2017	L	M	L	L	L	L	L

Figure S.23 Risk of bias assessment of studies investigating new-onset prediabetes using the QUIPS tool. L – low, M – moderate, H – high, NA – not applicable

Figure S.24 – Risk of bias assessment: diabetes

A) overall assessment

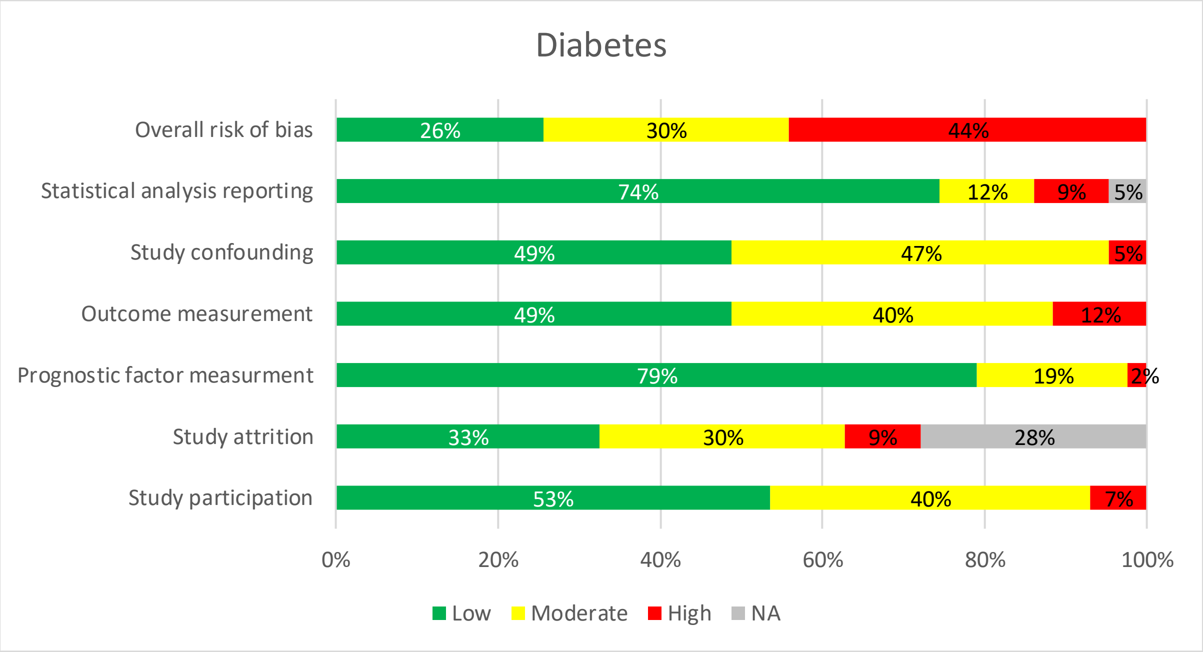

B) scoring of individual studies

Study	Study participation	Study attrition	Prognostic factor measurement	Outcome measurement	Study confounding	Statistical analysis reporting	Overall risk of bias
Andersson et al., 2010	L	L	M	L	M	L	M
Angelini et al., 1984	H	H	L	M	M	NA	H
Bharmal et al., 2022a	L	L	M	M	L	L	M
Bojková et al., 2016	M	NA	L	H	M	M	H
Boreham & Ammori, 2003	M	H	L	M	M	L	H
Burge & Gabaldon-Bates, 2003	H	NA	L	H	M	L	H
Buscher et al., 1999	L	M	L	L	M	L	M
Castoldi et al., 2013	L	NA	L	H	M	L	H
Chandrasekaran et al., 2015	L	L	L	L	M	L	L
Cho et al., 2020	L	NA	L	M	M	L	M
Cho et al., 2021	L	NA	L	M	M	L	M
Chowdhury et al., 2022	M	M	M	L	M	H	H
Doepel et al., 1993	M	L	L	M	L	M	H
Ermolov et al., 2016	M	M	L	M	M	L	H
Firkins et al., 2021	M	NA	M	M	L	L	H

Frey, 1969	M	NA	L	H	M	NA	H
Gold-Smith et al., 2020	L	NA	L	L	L	L	L
Halonen et al., 2003	M	L	L	M	M	L	H
Hietanen et al., 2014	L	M	L	M	L	L	M
Ho et al., 2015	L	NA	M	M	L	L	M
Hochman et al., 2006	M	L	L	M	L	L	M
Huang et al., 2022	L	L	M	L	L	M	M
Koziel et al., 2017	L	M	L	L	L	L	L
Lv et al., 2022	L	L	L	M	L	L	L
Ma et al., 2019	L	NA	L	L	L	L	L
Man et al., 2022	L	M	L	L	M	L	M
Miko et al., 2022b	M	H	M	L	M	H	H
Nikkola et al., 2017	M	L	L	L	L	L	L
Nikolic et al., 2022	M	L	L	L	L	L	L
Patra & Das, 2021	L	L	L	L	L	L	L
Robertson et al., 2023	L	M	L	L	L	L	L
Symersky et al., 2006	L	M	L	L	M	L	M
Takeyama, 2009	H	NA	L	L	M	M	H
Thiruvengadam et al., 2023	M	L	L	L	H	L	H
Trikudanathan et al., 2022	M	M	L	M	L	H	H
Tu et al., 2017	L	M	L	L	L	L	L
Tu et al., 2023	L	M	L	L	M	L	M
Uomo et al., 2010	L	L	L	M	M	M	H
Vujasinovic et al., 2014	M	H	L	L	L	L	H
Walker et al., 2022b	L	L	H	H	H	L	H
Wundsam et al., 2019	M	NA	L	M	L	L	M
Zhang et al., 2022	M	M	M	M	L	H	H
Zhang et al., 2023	L	M	L	L	L	L	L

Figure S.24 Risk of bias assessment of studies investigating new-onset diabetes using the QUIPS tool. L – low, M – moderate, H – high, NA – not applicable

Figure S.25 – Risk of bias assessment: prediabetes/diabetes

A) overall assessment

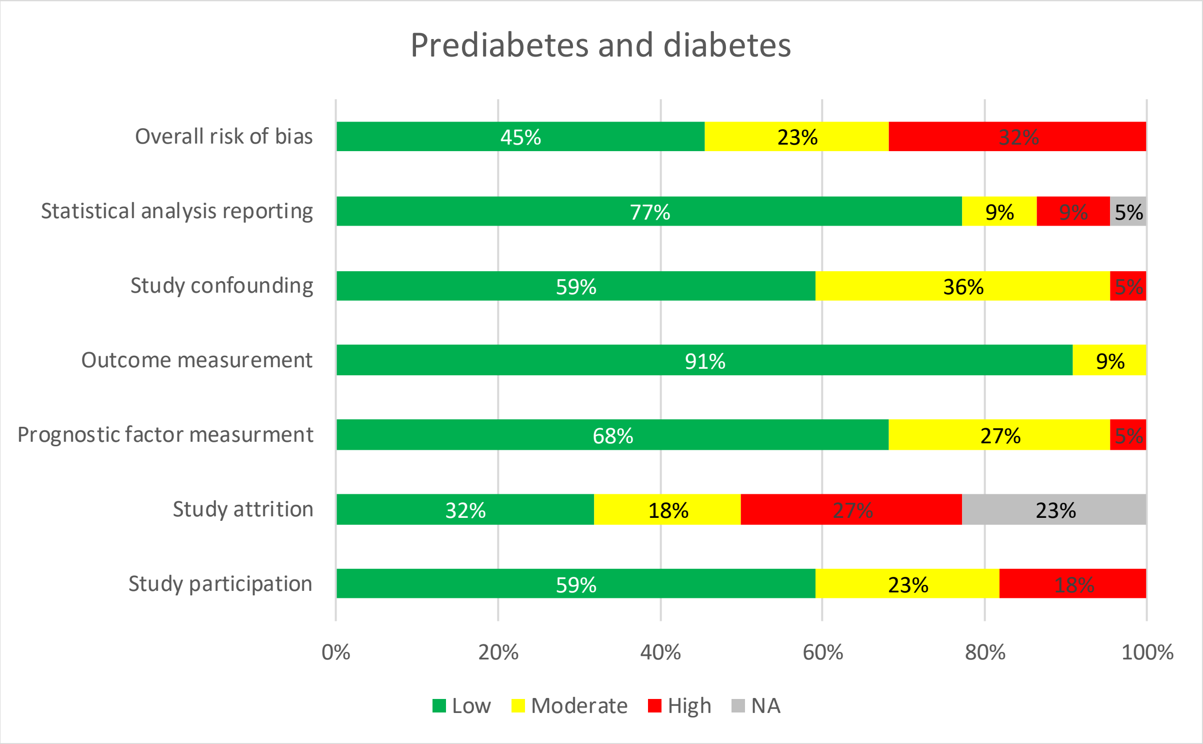

B) scoring of individual studies

Study	Study participation	Study attrition	Prognostic factor measurement	Outcome measurement	Study confounding	Statistical analysis reporting	Overall risk of bias
Akbar et al., 2020	H	H	M	M	M	H	H
Akbar et al., 2022	L	L	L	L	L	L	L
Andersson et al., 2010	L	L	M	L	M	L	M
Angelini et al., 1984	H	H	L	M	M	NA	H
Buscher et al., 1999	L	M	L	L	M	L	M
Garip et al., 2013	M	L	L	L	M	L	M
Guo et al., 2022	L	H	H	L	H	L	H
Li et al. 2021	L	NA	L	L	L	L	L
Malecka-Panas et al., 1998	H	H	L	L	L	M	H
Malecka-Panas et al., 2002	H	H	L	L	L	L	H
Miko et al., 2022b	M	H	M	L	M	H	H
Nikkola et al., 2017	M	L	L	L	L	L	L
Norbitt et al., 2021	L	L	M	L	L	L	L

Norbitt et al., 2022	M	L	M	L	L	L	M
Pendharkar et al., 2017b	L	NA	M	L	L	L	L
Pendharkar et al., 2018b	L	NA	L	L	L	L	L
Trgo et al., 2016	M	M	L	L	M	L	H
Tu et al., 2017	L	M	L	L	L	L	L
Tu et al., 2018	L	M	L	L	M	L	M
Wu et al., 2011	L	L	L	L	L	M	L
Yu et al., 2020	L	NA	L	L	L	L	L
Yuan et al., 2017	L	NA	L	L	L	L	L

Figure S.256 Risk of bias assessment of studies investigating new-onset prediabetes and diabetes using the QUIPS tool. L – low, M – moderate, H – high, NA – not applicable

RESULTS: Sensitivity analysis

Figure S.26 – Sensitivity analysis: severe AP

A)

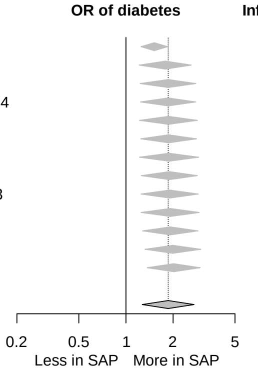

Study	Influental	effect size	95% CI	I2	Std residual	Dffits	Cook's dist.	Covariance ratio	Hat value
Omitting Ma et al., 2019	*	1.52	[1.25; 1.85]	0.53	4.08	1.59	1.36	0.26	0.07
Omitting Koziel et al., 2017		1.79	[1.22; 2.63]	0.71	1.16	0.24	0.06	1.00	0.04
Omitting Miko et al., 2022b		1.86	[1.23; 2.81]	0.72	0.31	0.01	0.00	1.15	0.04
Omitting Vujasinovic et al., 2014		1.86	[1.24; 2.80]	0.72	0.34	0.01	0.00	1.13	0.02
Omitting Lv et al., 2022		1.87	[1.22; 2.87]	0.72	0.10	-0.03	0.00	1.23	0.10
Omitting Man et al., 2022		1.88	[1.24; 2.83]	0.73	0.12	-0.04	0.00	1.15	0.03
Omitting Firkins et al., 2021		1.89	[1.22; 2.92]	0.66	-0.06	-0.08	0.01	1.28	0.14
Omitting Symersky et al., 2006		1.90	[1.26; 2.87]	0.73	-0.24	-0.11	0.01	1.15	0.04
Omitting Robertson et al., 2023		1.91	[1.25; 2.91]	0.73	-0.21	-0.12	0.02	1.22	0.09
Omitting Takeyama, 2009		1.92	[1.25; 2.95]	0.73	-0.31	-0.16	0.03	1.24	0.12
Omitting Castoldi et al., 2013		1.92	[1.28; 2.89]	0.72	-0.56	-0.17	0.03	1.13	0.05
Omitting Cho et al., 2020		2.00	[1.32; 3.01]	0.65	-1.02	-0.40	0.16	1.15	0.13
Omitting Ho et al., 2015		2.02	[1.37; 2.98]	0.62	-1.46	-0.50	0.21	1.03	0.13
**Random effect**		**1.86**	**[1.27; 2.73]**						
I^2 = 70% [47%; 83%]									

B)

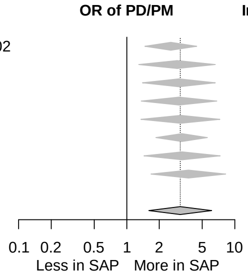

Study	Influental	effect size	95% CI	I2	Std residual	Dffits	Cook's dist.	Covariance ratio	Hat value
Omitting Malecka-Panas et al., 2002	*	2.55	[1.47; 4.44]	0.20	1.98	0.85	0.51	0.64	0.12
Omitting Garip et al., 2013		2.92	[1.29; 6.62]	0.46	0.55	0.22	0.06	1.39	0.17
Omitting Pendharkar et al., 2018b		3.04	[1.40; 6.62]	0.51	0.47	0.09	0.01	1.25	0.05
Omitting Andersson et al., 2010		3.05	[1.36; 6.84]	0.51	0.32	0.08	0.01	1.36	0.09
Omitting Tu et al., 2017		3.14	[1.35; 7.30]	0.52	0.03	-0.01	0.00	1.48	0.16
Omitting Yuan et al., 2017		3.22	[1.86; 5.58]	0.35	-1.90	-0.11	0.01	0.63	0.06
Omitting Miko et al., 2022b		3.26	[1.44; 7.35]	0.53	-0.29	-0.13	0.02	1.38	0.11
Omitting Yu et al., 2020		3.72	[1.68; 8.23]	0.27	-1.06	-0.62	0.37	1.31	0.25
**Random effect**		**3.13**	**[1.60; 6.11]**						
I^2 = 45% [0%; 76%]									

Figure S.267 Sensitivity analysis of the association between severe acute pancreatitis and new-onset A) diabetes and B) prediabetes and diabetes. OR – odds ratio, CI – confidence interval, AP – acute pancreatitis, SAP – severe AP, PD/DM – prediabetes and diabetes

Figure S.27 – Sensitivity analysis: severe or moderate AP

A)

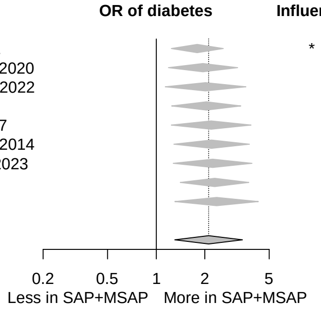

Study	Influental	effect size	95% CI	I2	Std residual	Dffits	Cook's dist.	Covariance ratio	Hat value
Omitting Patra & Das, 2021	*	1.79	[1.24; 2.60]	0.04	2.02	0.88	0.60	0.56	0.09
Omitting Gold-Smith et al., 2020		1.95	[1.19; 3.19]	0.26	1.20	0.39	0.15	0.99	0.08
Omitting Chowdhury et al., 2022		2.02	[1.14; 3.59]	0.32	0.59	0.19	0.04	1.36	0.16
Omitting Man et al., 2022		2.04	[1.25; 3.34]	0.32	1.06	0.16	0.03	0.99	0.02
Omitting Nikkola et al., 2017		2.19	[1.24; 3.86]	0.40	-0.22	-0.17	0.03	1.32	0.08
Omitting Vujasinovic et al., 2014		2.20	[1.29; 3.77]	0.38	-0.61	-0.20	0.04	1.18	0.04
Omitting Robertson et al., 2023		2.24	[1.28; 3.91]	0.39	-0.55	-0.26	0.08	1.29	0.10
Omitting Miko et al., 2022b		2.30	[1.41; 3.75]	0.28	-1.37	-0.44	0.17	0.98	0.14
Omitting Lv et al., 2022		2.36	[1.30; 4.29]	0.29	-0.82	-0.51	0.30	1.45	0.28
**Random effect**		**2.11**	**[1.30; 3.41]**						
I^2 = 31% [0%; 68%]									

B)

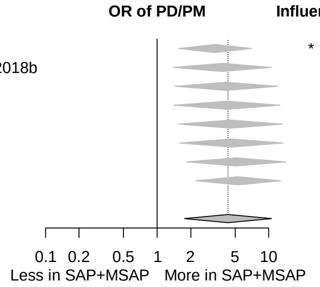

Study	Influental	effect size	95% CI	I2	Std residual	Dffits	Cook's dist.	Covariance ratio	Hat value
Omitting Trgo et al., 2016	*	3.31	[1.57; 6.98]	0.61	2.18	0.79	0.49	0.65	0.08
Omitting Pendharkar et al., 2018b		3.84	[1.40; 10.52]	0.68	0.88	0.30	0.09	1.17	0.12
Omitting Akbar et al., 2022		4.16	[1.45; 11.96]	0.72	0.43	0.09	0.01	1.29	0.11
Omitting Yu et al., 2020		4.24	[1.42; 12.61]	0.70	0.27	0.05	0.00	1.38	0.15
Omitting Tu et al., 2017		4.53	[1.54; 13.29]	0.73	-0.13	-0.12	0.02	1.34	0.11
Omitting Nikkola et al., 2017		4.63	[1.59; 13.46]	0.74	-0.29	-0.17	0.03	1.32	0.11
Omitting Miko et al., 2022b		5.10	[1.83; 14.23]	0.70	-0.95	-0.43	0.19	1.22	0.17
Omitting Yuan et al., 2017		5.31	[2.21; 12.75]	0.58	-1.78	-0.66	0.29	0.89	0.16
**Random effect**		**4.32**	**[1.76; 10.60]**						
I^2 = 69% [36%; 85%]									

Figure S.278 Sensitivity analysis of the association between severe or moderately severe acute pancreatitis and new-onset A) diabetes and B) prediabetes and diabetes. OR – odds ratio, CI – confidence interval, AP – acute pancreatitis, SAP – severe AP, MSAP – moderately severe AP, PD/DM – prediabetes and diabetes

Figure S.28 – Sensitivity analysis: necrosis

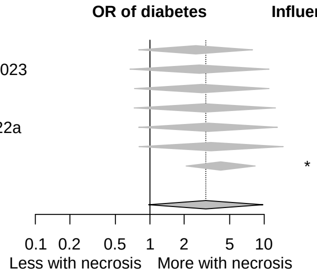

Study	Influental	effect size	95% CI	I2	Std residual	Dffits	Cook's dist.	Covariance ratio	Hat value
Omitting Tu et al., 2017		2.52	[0.80; 7.94]	0.75	1.44	0.45	0.19	0.91	0.07
Omitting Robertson et al., 2023		2.72	[0.67; 11.04]	0.72	0.65	0.25	0.07	1.35	0.18
Omitting Patra & Das, 2021		2.85	[0.73; 11.05]	0.77	0.59	0.16	0.03	1.26	0.11
Omitting Man et al., 2022		3.03	[0.73; 12.60]	0.78	0.25	0.04	0.00	1.40	0.15
Omitting Bharmal et al., 2022a		3.23	[0.80; 13.04]	0.79	-0.03	-0.09	0.01	1.34	0.10
Omitting Zhang et al., 2023		3.44	[0.80; 14.70]	0.79	-0.30	-0.21	0.05	1.45	0.19
Omitting Takeyama, 2009	*	4.17	[2.08; 8.37]	0.00	-4.37	-1.62	0.41	0.38	0.20
**Random effect**		**3.09**	**[0.98; 9.72]**						
I^2 = 74% [45%; 88%]									

Figure S.28 Sensitivity analysis of the association between pancreatic necrosis and new-onset diabetes. OR – odds ratio, CI – confidence interval

Figure S.29 – Sensitivity analysis: alcoholic AP

A)

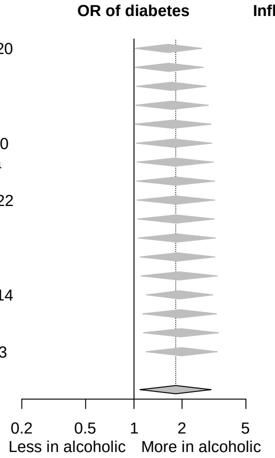

Study	Influental	effect size	95% CI	I2	Std residual	Dffits	Cook's dist.	Covariance ratio	Hat value
Omitting Gold-Smith et al., 2020		1.64	[1.01; 2.65]	0.88	1.83	0.47	0.19	0.87	0.05
Omitting Patra & Das, 2021		1.66	[1.01; 2.71]	0.88	1.62	0.42	0.16	0.93	0.05
Omitting Buscher et al., 1999		1.72	[1.05; 2.82]	0.89	1.47	0.25	0.06	0.93	0.03
Omitting Doepel et al., 1993		1.73	[1.03; 2.91]	0.89	1.05	0.22	0.05	1.03	0.04
Omitting Takeyama, 2009		1.75	[1.01; 3.04]	0.87	0.58	0.15	0.03	1.15	0.08
Omitting Andersson et al., 2010		1.78	[1.04; 3.07]	0.89	0.49	0.09	0.01	1.12	0.04
Omitting Bharmal et al., 2022a		1.81	[1.05; 3.13]	0.89	0.21	0.02	0.00	1.14	0.05
Omitting Ho et al., 2015		1.83	[1.04; 3.19]	0.81	0.06	-0.01	0.00	1.19	0.09
Omitting Chowdhury et al., 2022		1.83	[1.05; 3.19]	0.89	0.01	-0.02	0.00	1.17	0.07
Omitting Bojková et al., 2016		1.83	[1.06; 3.18]	0.89	-0.00	-0.02	0.00	1.15	0.05
Omitting Frey, 1969		1.85	[1.06; 3.23]	0.89	-0.15	-0.07	0.00	1.18	0.08
Omitting Angelini et al., 1984		1.87	[1.09; 3.21]	0.89	-0.51	-0.11	0.01	1.10	0.03
Omitting Ma et al., 2019		1.91	[1.11; 3.31]	0.89	-0.63	-0.20	0.04	1.14	0.08
Omitting Vujasinovic et al., 2014		1.93	[1.20; 3.10]	0.89	-1.84	-0.25	0.06	0.86	0.03
Omitting Lv et al., 2022		1.93	[1.14; 3.28]	0.89	-0.99	-0.24	0.06	1.06	0.06
Omitting Firkins et al., 2021		1.96	[1.15; 3.36]	0.65	-0.99	-0.31	0.10	1.10	0.09
Omitting Robertson et al., 2023		1.99	[1.19; 3.31]	0.89	-1.46	-0.38	0.13	0.99	0.07
**Random effect**		**1.82**	**[1.09; 3.04]**						
I^2 = 88% [83%; 92%]									

B)

Study	Influental	effect size	95% CI	I2	Std residual	Dffits	Cook's dist.	Covariance ratio	Hat value
Omitting Malecka-Panas et al., 2002	*	1.07	[0.72; 1.61]	0.00	2.59	1.03	0.87	0.78	0.11
Omitting Buscher et al., 1999		1.29	[0.70; 2.37]	0.25	0.75	0.14	0.02	1.15	0.05
Omitting Li et al. 2021		1.33	[0.68; 2.60]	0.30	0.25	0.02	0.00	1.40	0.14
Omitting Yuan et al., 2017		1.37	[0.70; 2.69]	0.31	-0.09	-0.12	0.02	1.39	0.12
Omitting Wu et al., 2011		1.40	[0.74; 2.64]	0.30	-0.44	-0.20	0.04	1.26	0.06
Omitting Angelini et al., 1984		1.41	[0.75; 2.66]	0.29	-0.59	-0.24	0.06	1.24	0.08
Omitting Pendharkar et al., 2018b		1.45	[0.77; 2.74]	0.27	-0.75	-0.34	0.13	1.26	0.14
Omitting Yu et al., 2020		1.55	[0.79; 3.08]	0.17	-0.99	-0.66	0.44	1.45	0.31
**Random effect**		**1.33**	**[0.77; 2.31]**						

I^2 = 20% [0%; 62%]

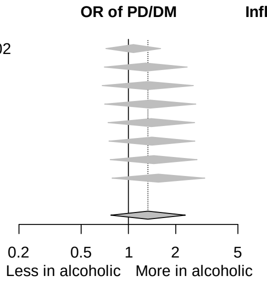

Figure S.99 Sensitivity analysis of the association between alcoholic acute pancreatitis and new-onset onset A) diabetes and B) prediabetes and diabetes. OR – odds ratio, CI – confidence interval, PD/DM – prediabetes and diabetes

Figure S.30 – Sensitivity analysis: biliary AP

A)

Study	Influental	effect size	95% CI	I2	Std residual	Dffits	Cook's dist.	Covariance ratio	Hat value
Omitting Robertson et al., 2023		0.67	[0.51; 0.88]	0.75	1.83	0.38	0.12	0.81	0.07
Omitting Cho et al., 2020		0.67	[0.49; 0.92]	0.71	0.99	0.33	0.11	1.12	0.10
Omitting Firkins et al., 2021		0.68	[0.49; 0.96]	0.73	0.50	0.19	0.04	1.23	0.10
Omitting Frey, 1969		0.69	[0.49; 0.95]	0.77	0.52	0.17	0.03	1.18	0.07
Omitting Bharmal et al., 2022a		0.69	[0.50; 0.94]	0.77	0.88	0.16	0.02	1.06	0.03
Omitting Ma et al., 2019		0.69	[0.49; 0.96]	0.77	0.34	0.13	0.02	1.23	0.09
Omitting Vujasinovic et al., 2014		0.69	[0.53; 0.90]	0.76	1.90	0.10	0.01	0.74	0.01
Omitting Takeyama, 2009		0.70	[0.50; 0.99]	0.77	-0.20	-0.02	0.00	1.22	0.07
Omitting Bojková et al., 2016		0.71	[0.51; 0.98]	0.77	-0.32	-0.03	0.00	1.16	0.03
Omitting Andersson et al., 2010		0.71	[0.51; 0.98]	0.77	-0.52	-0.06	0.00	1.13	0.02
Omitting Boreham & Ammori, 2003		0.71	[0.52; 0.97]	0.76	-0.98	-0.11	0.01	1.02	0.01
Omitting Buscher et al., 1999		0.72	[0.53; 0.97]	0.76	-1.11	-0.13	0.02	0.98	0.01
Omitting Lv et al., 2022		0.72	[0.51; 1.00]	0.76	-0.52	-0.14	0.02	1.20	0.09
Omitting Chowdhury et al., 2022		0.72	[0.52; 1.00]	0.76	-0.65	-0.15	0.02	1.15	0.06
Omitting Walker et al., 2022b		0.72	[0.51; 1.00]	0.75	-0.56	-0.15	0.03	1.21	0.10
Omitting Man et al., 2022		0.72	[0.53; 0.98]	0.76	-1.02	-0.17	0.03	1.02	0.03
Omitting Hochman et al., 2006		0.72	[0.56; 0.94]	0.75	-1.95	-0.21	0.04	0.72	0.01
Omitting Ho et al., 2015		0.72	[0.52; 1.01]	0.63	-0.68	-0.21	0.05	1.19	0.10
**Random effect**		**0.70**	**[0.52; 0.95]**						

I^2 = 75% [61%; 84%]

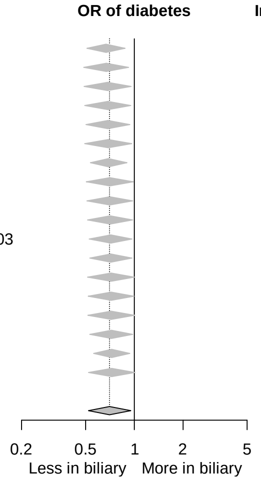

B)

Study	Influental	effect size	95% CI	I2	Std residual	Dffits	Cook's dist.	Covariance ratio	Hat value
Omitting Li et al. 2021		0.68	[0.51; 0.91]	0	1.21	0.36	0.13	1.09	0.08
Omitting Yu et al., 2020	*	0.69	[0.46; 1.04]	0	0.33	0.29	0.08	1.76	0.43
Omitting Pendharkar et al., 2018b		0.70	[0.51; 0.96]	0	0.57	0.18	0.03	1.10	0.09
Omitting Buscher et al., 1999		0.72	[0.53; 0.99]	0	-0.09	-0.01	0.00	1.03	0.02
Omitting Malecka-Panas et al., 2002		0.73	[0.54; 1.00]	0	-0.62	-0.14	0.02	1.05	0.05
Omitting Wu et al., 2011		0.75	[0.56; 1.00]	0	-1.22	-0.29	0.09	1.06	0.05
Omitting Yuan et al., 2017		0.75	[0.53; 1.07]	0	-0.54	-0.32	0.10	1.35	0.26
**Random effect**		**0.72**	**[0.55; 0.95]**						

I^2 = 0% [0%; 71%]

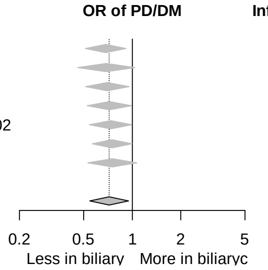

Figure S.30 Sensitivity analysis of the association between biliary acute pancreatitis and new-onset A) diabetes and B) prediabetes and diabetes. OR – odds ratio, CI – confidence interval, PD/DM – prediabetes and diabetes

Figure S.31 – Sensitivity analysis: recurrent AP

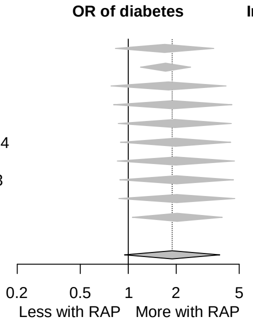

Study	Influental	effect size	95% CI	I2	Std residual	Dffits	Cook's dist.	Covariance ratio	Hat value
Omitting Tu et al., 2023		1.69	[0.83; 3.45]	0.78	1.14	0.37	0.13	1.02	0.09
Omitting Nikkola et al., 2017		1.72	[1.19; 2.47]	0.76	2.57	0.34	0.10	0.27	0.03
Omitting Lv et al., 2022		1.79	[0.78; 4.13]	0.74	0.46	0.16	0.03	1.40	0.14
Omitting Ho et al., 2015		1.90	[0.81; 4.49]	0.71	0.02	-0.02	0.00	1.48	0.15
Omitting Man et al., 2022		1.97	[0.87; 4.46]	0.80	-0.30	-0.12	0.02	1.35	0.07
Omitting Vujasinovic et al., 2014		1.99	[0.89; 4.41]	0.80	-0.49	-0.16	0.03	1.28	0.06
Omitting Cho et al., 2020		1.99	[0.85; 4.65]	0.80	-0.32	-0.15	0.03	1.45	0.15
Omitting Robertson et al., 2023		2.01	[0.88; 4.59]	0.80	-0.46	-0.19	0.04	1.37	0.11
Omitting Firkins et al., 2021		2.02	[0.87; 4.69]	0.63	-0.43	-0.20	0.05	1.42	0.15
Omitting Bharmal et al., 2022a		2.03	[1.06; 3.91]	0.79	-1.35	-0.25	0.06	0.86	0.05
**Random effect** I^2 = 78% [60%; 88%]		**1.89**	**[0.95; 3.77]**						

Figure S.31 Sensitivity analysis of the association between recurrent acute pancreatitis and new-onset diabetes. OR – odds ratio, CI – confidence interval, RAP – recurrent acute pancreatitis

Figure S.32 – Sensitivity analysis: smoking

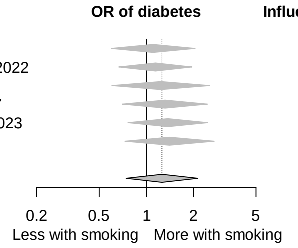

Study	Influental	effect size	95% CI	I2	Std residual	Dffits	Cook's dist.	Covariance ratio	Hat value
Omitting Cho et al., 2021		1.10	[0.60; 2.04]	0.67	1.23	0.68	0.41	1.17	0.23
Omitting Chowdhury et al., 2022		1.14	[0.67; 1.96]	0.95	1.47	0.51	0.22	0.90	0.14
Omitting Lv et al., 2022		1.24	[0.60; 2.53]	0.95	0.09	0.07	0.01	1.59	0.19
Omitting Nikkola et al., 2017		1.31	[0.70; 2.45]	0.95	-0.75	-0.22	0.05	1.21	0.07
Omitting Robertson et al., 2023		1.37	[0.76; 2.46]	0.95	-1.14	-0.43	0.18	1.06	0.13
Omitting Firkins et al., 2021		1.40	[0.73; 2.70]	0.66	-0.97	-0.54	0.30	1.33	0.24
**Random effect** I^2 = 94% [89%; 97%]		**1.26**	**[0.74; 2.13]**						

Figure S.32 Sensitivity analysis of the association between smoking and new-onset diabetes. OR – odds ratio, CI – confidence interval

Figure S.33 – Sensitivity analysis: obesity

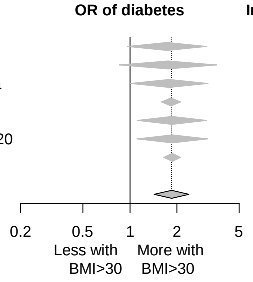

Study	Influental	effect size	95% CI	I2	Std residual	Dffits	Cook's dist.	Covariance ratio	Hat value
Omitting Zhang et al., 2023		1.73	[0.96; 3.11]	0.16	0.40	0.48	0.38	4.78	0.09
Omitting Firkins et al., 2021	*	1.76	[0.86; 3.60]	0.18	0.12	0.17	0.23	7.18	0.81
Omitting Bharmal et al., 2022a		1.78	[1.01; 3.16]	0.18	0.10	0.30	0.11	4.54	0.02
Omitting Walker et al., 2022b		1.83	[1.59; 2.11]	0.00	1.51	0.06	0.00	0.36	0.03
Omitting Man et al., 2022		1.86	[1.11; 3.10]	0.13	-0.54	-0.06	0.00	3.67	0.02
Omitting Gold-Smith et al., 2020		1.87	[1.11; 3.15]	0.13	-0.53	-0.09	0.01	3.81	0.03
Omitting Nikkola et al., 2017		1.87	[1.65; 2.12]	0.00	-1.74	-0.10	0.01	0.35	0.02
**Random effect** I^2 = 2% [0%; 71%]		**1.85**	**[1.43; 2.38]**						

Figure S.33 Sensitivity analysis of the association between obesity and new-onset diabetes. OR – odds ratio, CI – confidence interval, BMI – body mass index

Figure S.34 – Sensitivity analysis: male sex

A)

Study	Influental	effect size	95% CI	I2	Std residual	Dffits	Cook's dist.	Covariance ratio	Hat value
Omitting Chowdhury et al., 2022		1.33	[0.84; 2.11]	0.63	0.86	0.29	0.09	1.16	0.09
Omitting Walker et al., 2022b		1.33	[0.82; 2.17]	0.60	0.63	0.26	0.08	1.31	0.14
Omitting Ho et al., 2015		1.34	[0.82; 2.19]	0.57	0.57	0.24	0.07	1.34	0.15
Omitting Bharmal et al., 2022a		1.38	[0.94; 2.01]	0.62	1.50	0.12	0.01	0.80	0.02
Omitting Vujasinovic et al., 2014		1.38	[0.88; 2.15]	0.64	0.79	0.11	0.01	1.10	0.01
Omitting Lv et al., 2022		1.40	[0.84; 2.32]	0.64	0.01	0.03	0.00	1.41	0.13
Omitting Man et al., 2022		1.40	[0.86; 2.27]	0.65	0.02	0.03	0.00	1.30	0.05
Omitting Cho et al., 2020		1.41	[0.84; 2.34]	0.61	-0.05	0.00	0.00	1.43	0.15
Omitting Burge & Gabaldon-Bates, 2003		1.43	[0.86; 2.36]	0.63	-0.24	-0.07	0.01	1.39	0.13
Omitting Boreham & Ammori, 2003		1.43	[0.90; 2.27]	0.64	-0.54	-0.08	0.01	1.19	0.03
Omitting Uomo et al., 2010		1.47	[1.05; 2.06]	0.59	-1.90	-0.23	0.05	0.63	0.01
Omitting Robertson et al., 2023	*	1.59	[1.23; 2.06]	0.42	-2.71	-0.93	0.42	0.37	0.10
**Random effect**		**1.41**	**[0.92; 2.14]**						

I^2 = 61% [27%; 79%]

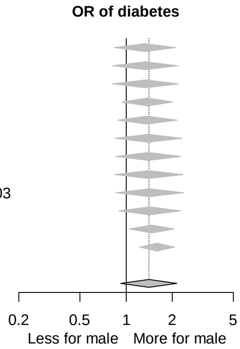

B)

Study	Influental	effect size	95% CI	I2	Std residual	Dffits	Cook's dist.	Covariance ratio	Hat value
Omitting Guo et al., 2022	*	1.12	[0.77; 1.65]	0.00	1.61	1.20	1.09	1.19	0.31
Omitting Akbar et al., 2022		1.25	[0.86; 1.84]	0.19	1.04	0.23	0.05	1.03	0.05
Omitting Li et al. 2021		1.28	[0.83; 1.97]	0.31	0.46	0.10	0.01	1.32	0.07
Omitting Pendharkar et al., 2018b		1.28	[0.82; 2.00]	0.32	0.33	0.06	0.00	1.37	0.08
Omitting Yuan et al., 2017		1.40	[0.97; 2.04]	0.00	-1.45	-0.65	0.35	0.99	0.20
Omitting Yu et al., 2020	*	1.43	[0.93; 2.21]	0.09	-1.21	-0.80	0.56	1.31	0.30
**Random effect**		**1.29**	**[0.91; 1.83]**						

I^2 = 18% [0%; 62%]

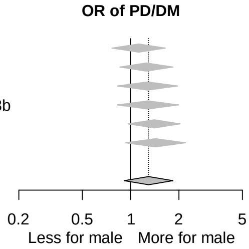

Figure S.34 Sensitivity analysis of the association between male sex and new-onset A) diabetes and B) prediabetes and diabetes. OR – odds ratio, CI – confidence interval, PD/DM – prediabetes and diabetes

RESULTS: Publication bias

Figure S.35 – Publication bias assessment: severe AP

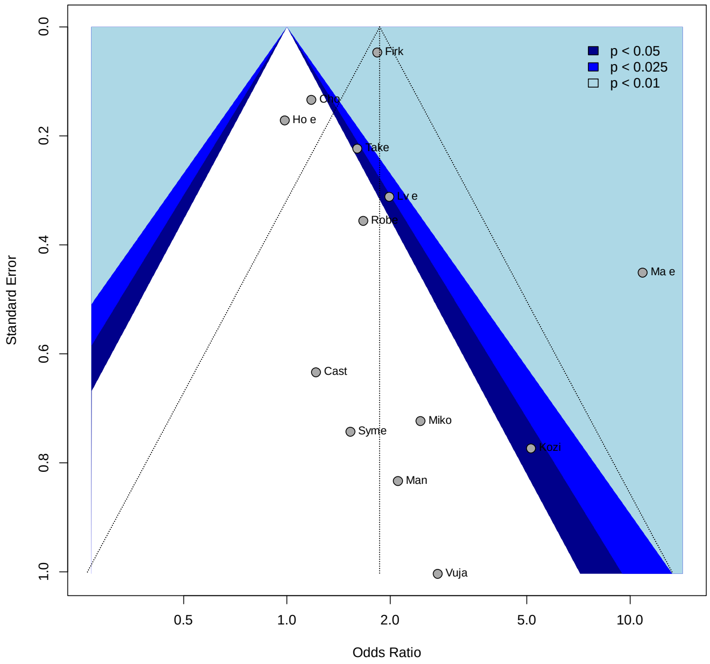

Figure S.35 Publication bias assessment of studies reporting on severe acute pancreatitis and new-onset diabetes. Egger's test p=0.8011

Figure S.36 – Publication bias assessment: severe or moderate AP

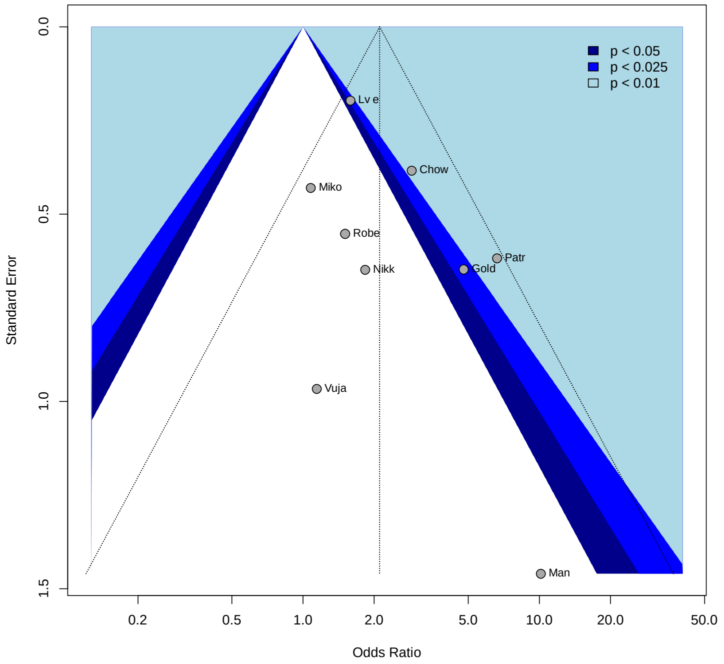

Figure S.36 Publication bias assessment of studies reporting on severe or moderately severe acute pancreatitis and new-onset diabetes. Egger's test p=0.2043

Figure S.37 – Publication bias assessment: alcoholic AP

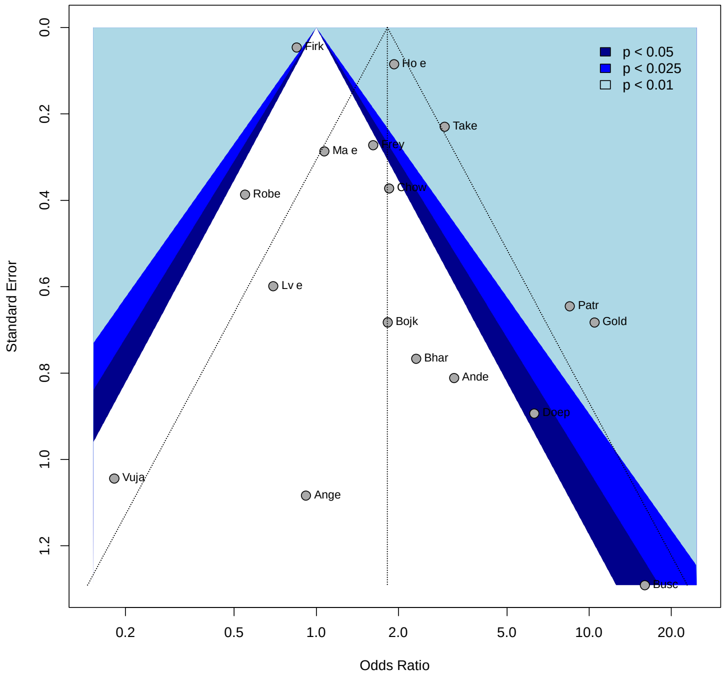

Figure S.37 Publication bias assessment of studies reporting on alcoholic etiology and new-onset diabetes. Egger's test p=0.0689

Figure S.38 – Publication bias assessment: biliary AP

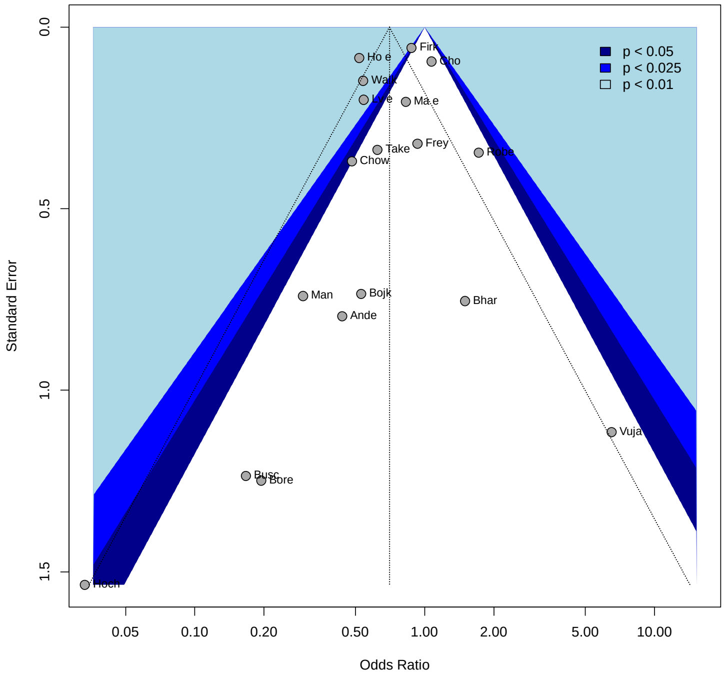

Figure S.38 Publication bias assessment of studies reporting on biliary etiology and new-onset diabetes. Egger's test p=0.3909

Figure S.39 – Publication bias assessment: recurrent AP

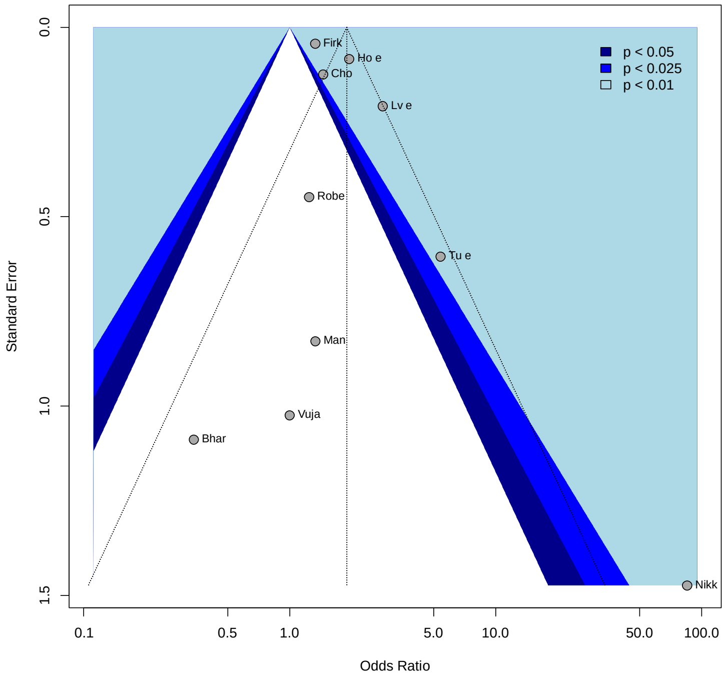

Figure S.39 Publication bias assessment of studies reporting on recurrent acute pancreatitis and new-onset diabetes. Egger's test p=0.2322

Figure S.40 – Publication bias assessment: male sex

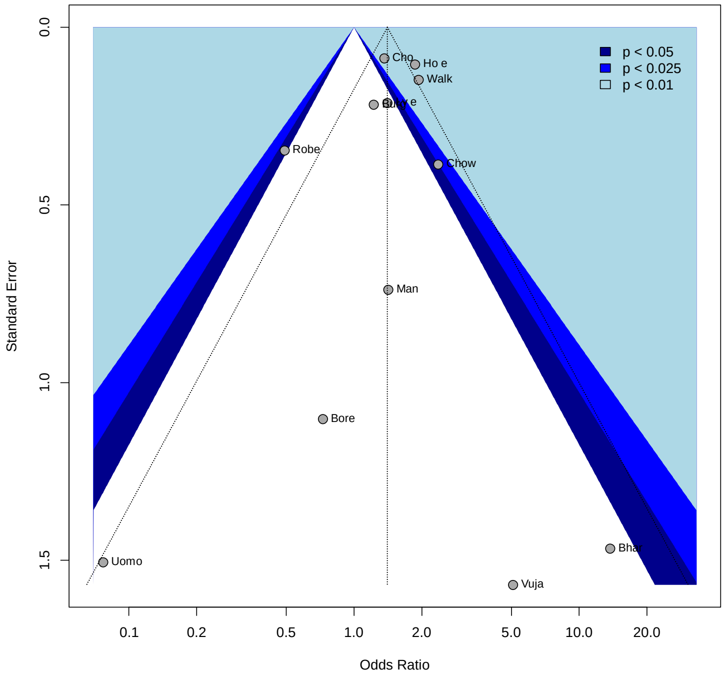

Figure S.40 Publication bias assessment of studies reporting on male sex and new-onset diabetes. Egger's test p=0.633

REFERENCES

1. EndNote [64 bit]. Version X9. Philadelphia, PA: Clarivate; 2013.

2. Cohen J. A coefficient of agreement for nominal scales. Educational Psychological Measurement 1960;20:37-46.

3. Microsoft Excel [Redmond; 2018.

4. Mantel N, Haenszel W. Statistical Aspects of the Analysis of Data From Retrospective Studies of Disease. JNCI: Journal of the National Cancer Institute 1959;22:719-748.

5. Robins J, Greenland S, Breslow NE. A general estimator for the variance of the Mantel-Haenszel odds ratio. Am J Epidemiol 1986;124:719-23.

6. Thompson SG, Turner RM, Warn DE. Multilevel models for meta-analysis, and their application to absolute risk differences. Statistical Methods in Medical Research 2001;10:375-392.

7. Cooper HM, Hedges LV, Valentine JC. The Handbook of Research Synthesis and Meta-Analysis. 2 ed. New York: Russell Sage Foundation; 2009.

8. Sweeting MJ, Sutton AJ, Lambert PC. What to add to nothing? Use and avoidance of continuity corrections in meta-analysis of sparse data. Stat Med 2004;23:1351-75.

9. IntHout J, Ioannidis JPA, Borm GF. The Hartung-Knapp-Sidik-Jonkman method for random effects meta-analysis is straightforward and considerably outperforms the standard DerSimonian-Laird method. BMC Medical Research Methodology 2014;14:25.

10. Knapp G, Hartung J. Improved tests for a random effects meta-regression with a single covariate. Stat Med 2003;22:2693-710.

11. Paule RC, Mandel J. Consensus Values and Weighting Factors. J Res Natl Bur Stand (1977) 1982;87:377-385.

12. Veroniki AA, Jackson D, Viechtbauer W, et al. Methods to estimate the between-study variance and its uncertainty in meta-analysis. Res Synth Methods 2016;7:55-79.

13. Higgins JP, Thompson SG. Quantifying heterogeneity in a meta-analysis. Stat Med 2002;21:1539-58.

14. Cochrane Handbook for Systematic Reviews of Interventions. Higgins J, Thomas J, Chandler J, et al. [insert producer], producer. Edition ed. Cochrane; 2015.

15. Doing meta-analysis with R: a hands-on guide. Harrer M, Cuijpers P, Furukawa T, et al. [insert producer], producer. Edition ed. Chapman & Hall/CRC Press; 2021.

16. Viechtbauer W, Cheung MWL. Outlier and influence diagnostics for meta-analysis. Research Synthesis Methods 2010;1:112-125.

17. Harbord RM, Egger M, Sterne JA. A modified test for small-study effects in meta-analyses of controlled trials with binary endpoints. Stat Med 2006;25:3443-57.

18. Das SL, Singh PP, Phillips AR, et al. Newly diagnosed diabetes mellitus after acute pancreatitis: a systematic review and meta-analysis. Gut 2014;63:818-831.

19. R: A language and environment for statistical computing [Vienna, Austria: R Foundation for Statistical Computing; 2022.

20. General Package for Meta-Analysis [2022.

21. Dmetar: Companion r Package for the Guide Doing Meta-Analysis in R [2022.

22. Yuan L, Tang M, Huang L, et al. Risk Factors of Hyperglycemia in Patients After a First Episode of Acute Pancreatitis: A Retrospective Cohort. Pancreas 2017;46:209-218.

23. Lv Y, Zhang J, Yang T, et al. Non-Alcoholic Fatty Liver Disease (NAFLD) Is an Independent Risk Factor for Developing New-Onset Diabetes After Acute Pancreatitis: A Multicenter Retrospective Cohort Study in Chinese Population. Frontiers in Endocrinology 2022;13:903731.

24. Ma JH, Yuan YJ, Lin SH, et al. Nomogram for predicting diabetes mellitus after the first attack of acute pancreatitis. Eur J Gastroenterol Hepatol 2019;31:323-328.

25. Guo SY, Yang HY, Ning XY, et al. Combination of Body Mass Index and Fasting Blood Glucose Improved Predictive Value of New-Onset Prediabetes or Diabetes After Acute Pancreatitis: A Retrospective Cohort Study. Pancreas 2022;51:388-393.

26. Zhang J, Lv Y, Li L. Stress hyperglycaemia is associated with an increased risk of postacute pancreatitis diabetes. Diabetologia 2022;65:S185-S186.

27. Zhang J, Lv Y, Hou J, et al. Machine learning for post-acute pancreatitis diabetes mellitus prediction and personalized treatment recommendations. Scientific Reports 2023;13:4857.

28. Huang J, Xu G, Ni M, et al. Long-term efficacy of endoscopic transluminal drainage for acute pancreatitis complicated with walled-off necrosis or pancreatic pseudocyst. Chinese Journal of Digestive Endoscopy 2022;39:128-132.

29. Nikkola J, Laukkarinen J, Lahtela J, et al. The Long-term Prospective Follow-up of Pancreatic Function After the First Episode of Acute Alcoholic Pancreatitis: Recurrence Predisposes One to Pancreatic Dysfunction and Pancreatogenic Diabetes. J Clin Gastroenterol 2017;51:183-190.

30. Angelini G, Pederzoli P, Caliari S, et al. Long-term outcome of acute necrohemorrhagic pancreatitis. A 4-year follow-up. Digestion 1984;30:131-7.

31. Bharmal SH, Pendharkar SA, Singh RG, et al. Associations between ketone bodies and fasting plasma glucose in individuals with post-pancreatitis prediabetes. Archives of Physiology and Biochemistry 2020;126:308-319.

32. Buscher HC, Jacobs ML, Ong GL, et al. Beta-cell function of the pancreas after necrotizing pancreatitis. Dig Surg 1999;16:496-500.

33. Bharmal SH, Kimita W, Ko J, et al. Cytokine signature for predicting new-onset prediabetes after acute pancreatitis: A prospective longitudinal cohort study. Cytokine 2022;150:155768.

34. Cho J, Scragg R, Petrov MS. The influence of cholecystectomy and recurrent biliary events on the risk of post-pancreatitis diabetes mellitus: a nationwide cohort study in patients with first attack of acute pancreatitis. HPB (Oxford) 2021;23:937-944.

35. Gold-Smith FD, Singh RG, Petrov MS. Elevated Circulating Levels of Motilin are Associated with Diabetes in Individuals after Acute Pancreatitis. Exp Clin Endocrinol Diabetes 2020;128:43-51.

36. Li X, Kimita W, Cho J, et al. Dietary Fibre Intake in Type 2 and New-Onset Prediabetes/Diabetes after Acute Pancreatitis: A Nested Cross-Sectional Study. Nutrients 2021;13.

37. Pendharkar SA, Singh RG, Petrov MS. Pro-inflammatory cytokine-induced lipolysis after an episode of acute pancreatitis. Arch Physiol Biochem 2018;124:401-409.

38. Pendharkar SA, Asrani VM, Murphy R, et al. The Role of Gut-brain Axis in Regulating Glucose Metabolism After Acute Pancreatitis. Clin Transl Gastroenterol 2017;8:e210.

39. Robertson FP, Lim W, Ratnayake B, et al. The development of new onset post-pancreatitis diabetes mellitus during hospitalisation is not associated with adverse outcomes. HPB (Oxford) 2023;25:1047-1055.

40. Wu D, Xu Y, Zeng Y, et al. Endocrine pancreatic function changes after acute pancreatitis. Pancreas 2011;40:1006-11.

41. Akbar W, Unnisa M, Tandan M, et al. New-onset prediabetes, diabetes after acute pancreatitis: A prospective cohort study with 12-month follow-up. Indian J Gastroenterol 2022;41:558-566.

42. Tu J, Zhang J, Ke L, et al. Endocrine and exocrine pancreatic insufficiency after acute pancreatitis: long-term follow-up study. BMC Gastroenterol 2017;17:114.

43. Miko A, Lillik V, Kato D, et al. Endocrine and exocrine insufficiency after a 2-year follow-up of acute pancreatitis: preliminary results of the GOULASH-PLUS study. Pancreatology 2022;22:e3.

44. Firkins SA, Hart PA, Papachristou GI, et al. Identification of a Risk Profile for New-Onset Diabetes After Acute Pancreatitis. Pancreas 2021;50:696-703.

45. Walker A, O'Kelly J, Graham C, et al. Increased risk of type 3c diabetes mellitus after acute pancreatitis warrants a personalized approach including diabetes screening. BJS Open 2022;6:zrac148.

46. Halonen KI, Pettilä V, Leppäniemi AK, et al. Long-term health-related quality of life in survivors of severe acute pancreatitis. Intensive Care Med 2003;29:782-6.

47. Tu J, Yang Y, Zhang J, et al. Effect of the disease severity on the risk of developing new-onset diabetes after acute pancreatitis. Medicine (Baltimore) 2018;97:e10713.

48. Yu BJ, Li NS, He WH, et al. Pancreatic necrosis and severity are independent risk factors for pancreatic endocrine insufficiency after acute pancreatitis: A long-term follow-up study. World J Gastroenterol 2020;26:3260-3270.

49. Man T, Seicean R, Lucaciu L, et al. Risk factors for new-onset diabetes mellitus following acute pancreatitis: a prospective study. European Review for Medical & Pharmacological Sciences 2022;26:5745-5754.

50. Ermolov AS, Blagovestnov DA, Rogal ML, et al. [Long-term results of severe acute pancreatitis management]. Khirurgiia (Mosk) 2016:11-15.

51. Chandrasekaran P, Gupta R, Shenvi S, et al. Prospective comparison of long term outcomes in patients with severe acute pancreatitis managed by operative and non operative measures. Pancreatology 2015;15:478-484.

52. Cho J, Dalbeth N, Petrov MS. Relationship between Gout and Diabetes Mellitus after Acute Pancreatitis: A Nationwide Cohort Study. J Rheumatology 2020;47:917-923.

53. Wundsam HV, Spaun GO, Bräuer F, et al. Evolution of Transluminal Necrosectomy for Acute Pancreatitis to Stent in Stent Therapy: Step-Up Approach Leads to Low Mortality and Morbidity Rates in 302 Consecutive Cases of Acute Pancreatitis. J Laparoendosc Adv Surg Tech A 2019;29:891-899.

54. Małecka-Panas E, Juszyński A, Gąsiorowska A, et al. Late outcome of pancreatic pseudocysts-a complication of acute pancreatitis induced by alcohol. Medical Science Monitor 1998;4:CR465-CR472.

55. Ho TW, Wu JM, Kuo TC, et al. Change of Both Endocrine and Exocrine Insufficiencies After Acute Pancreatitis in Non-Diabetic Patients: A Nationwide Population-Based Study. Medicine (Baltimore) 2015;94:e1123.

56. Burge MR, Gabaldon-Bates J. The Role of Ethnicity in Post-Pancreatitis Diabetes Mellitus. Diabetes Technology & Therapeutics 2003;5:183-188.

57. Chowdhury A, Kong N, Sun Kim J, et al. Predictors of Diabetes Mellitus Following Admission for Acute Pancreatitis: Analysis From a Prospective Observational Cohort. American Journal of Gastroenterology 2022;117:S4-S5.

58. Norbitt CF, Kimita W, Bharmal SH, et al. Relationship between Habitual Intake of Vitamins and New-Onset Prediabetes/Diabetes after Acute Pancreatitis. Nutrients 2022;14:1480.

59. Thiruvengadam NR, Schaubel DE, Forde KA, et al. Association of Statin Usage and the Development of Diabetes Mellitus after Acute Pancreatitis. Clinical Gastroenterology and Hepatology 2023;21:1214-1222.e14.

60. Trikudanathan G, Abdallah MA, Munigala S, et al. PREDICTORS FOR NEW ONSET DIABETES (NOD) FOLLOWING NECROTIZING PANCREATITIS (NP)- A SINGLE TERTIARY CENTER EXPERIENCE IN 525 PATIENTS. Gastroenterology 2022;162:S-175-S-176.

61. Nikolic S, Lanzillotta M, Panic N, et al. Unraveling the relationship between autoimmune pancreatitis type 2 and inflammatory bowel disease: Results from two centers and systematic review of the literature. United European Gastroenterology Journal 2022;10:496-506.
